# *Prunella vulgaris* L. – A Review of its Ethnopharmacology, Phytochemistry, Quality Control and Pharmacological Effects

**DOI:** 10.3389/fphar.2022.903171

**Published:** 2022-06-23

**Authors:** Junying Pan, Haoyu Wang, Yinghua Chen

**Affiliations:** First Affiliated Hospital, Heilongjiang University of Chinese Medicine, Harbin, China

**Keywords:** *Prunella vulgaris* L., chemical constituents, pharmacological effects, ethnopharmacology, traditional applications, analytical methods, quality control

## Abstract

*Prunella vulgaris* L. (PVL) is dried fruit spike of Lamiacea plant *Prunella vulgaris* L., which is a perennial herb with medicinal and edible homology used for thousands of years. PVL is bitter, acrid, cold, and belongs to the liver and gallbladder meridians. It clears the liver and dissipate fire, improve vision, disperse swelling, and has satisfactory clinical therapeutic effects on many diseases such as photophobia, dizziness, scrofula, goiter, breast cancer. The collection of information and data related to PVL comes from literatures retrieved and collated from various online scientific databases (such as CNKI, VIP, PubMed, Web of Science, Research Gate, Science Database), ancient books of traditional chinese medicine (Encyclopedia of Traditional Chinese Medicine, Classics of Traditional Chinese Medicine, Dictionary of Traditional Chinese Medicine), and Doctoral and Master’s Dissertations. Currently, the major chemical constituents isolated and identified from PVL are triterpenoids, steroids, flavonoids, phenylpropanoids, organic acids, volatile oils and polysaccharides. Modern pharmacological studies have shown that PVL has a wide range of pharmacological activities, including anti-inflammatory, anti-tumor, antibacterial and antiviral effects, as well as immune regulation, antihypertensive, hypoglycemic, lipid-lowering, antioxidant, free radical scavenging, liver protection, sedative and hypnotic effects. This paper reviewes the botany, ethnopharmacology, traditional application, phytochemistry, analytical methods, quality control, pharmacological effects of PVL. It can be used not only as medicine, but also gradually integrated into the “medicine and food homology” and “Chinese medicine health” boom. More importantly, it has great potential for drug resources development. This paper deeply discusses the shortcomings of current PVL research, and proposes corresponding solutions, in order to find a breakthrough point for PVL research in the future. At the same time, it is necessary to further strengthen the research on its medicinal chemistry, mechanism of action and clinical application efficacy in the future, and strive to extract, purify and synthesize effective components with high efficiency and low toxicity, so as to improve the safety and rationality of clinical medication.

## Introduction

**FIGURE 1:**
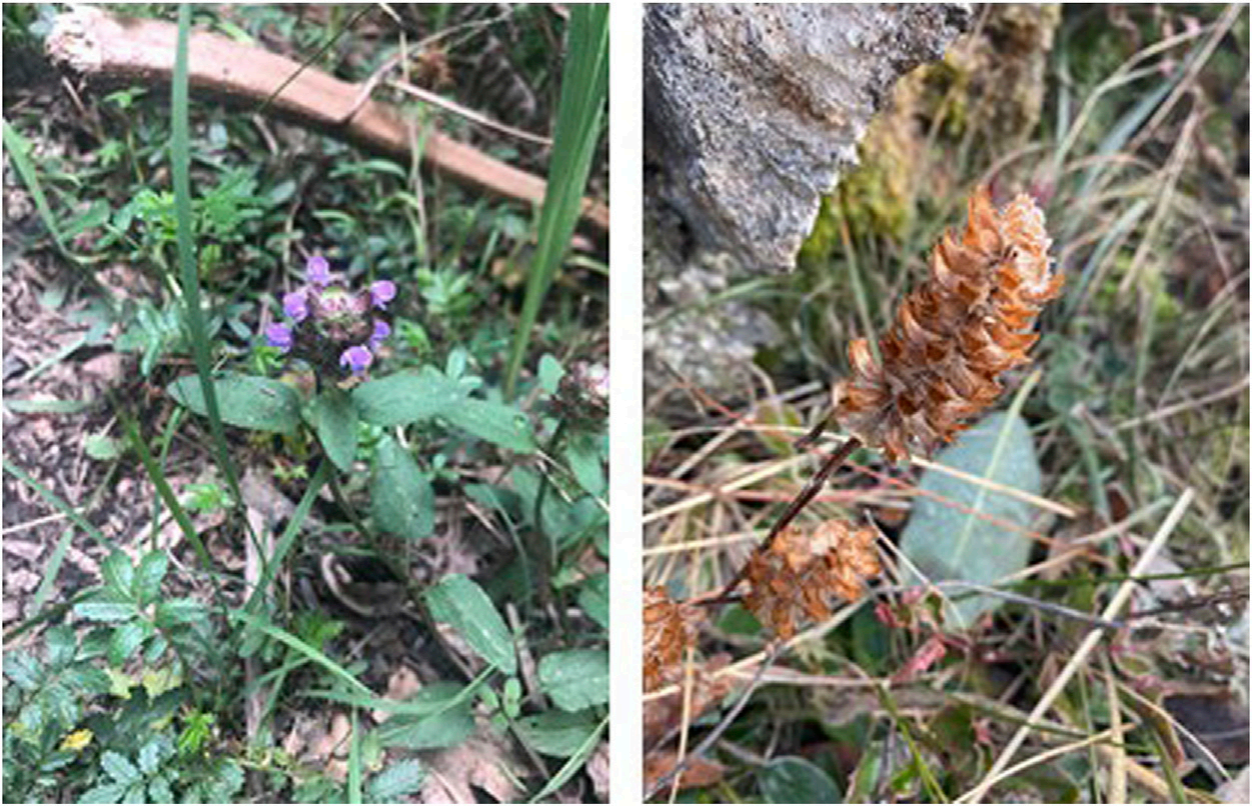
Aerial parts and dry spikes of *Prunella vulgaris L.* (Global Biodiversity Information Facility).

PVL (Figure 1) is the dried fruit spike of Lamiaceae plant *Prunella vulgaris* L., widely distributed in the Eurasian temperate and tropical regions, Northwest Africa and North America (Jian et al.). In China, the records of PVL originated from “Shen Nong’s Classic of the Materia Medica” (Shén Nóng Bĕn Căo Jīng, 神农本草经) (Dong Han Dynasty, AD 25-220). It is nominated because of its characteristics of flowering in spring and withering after summer. There are many synonyms when it comes to PVL such as Xiju, Naidong, Yanmian, Tiesecao, Datouhua. As a folk medicine used for thousands of years in China, PVL is mainly used for relieving sore throat, antipyretic and accelerating wound healing (Rasool and Ganai, 2013). In March 2010, National Health Commission of the People’s Republic of China announced the inclusion of PVL as one of the new Chinese herbal medicines in the list of medicinal and food homology, and stipulated that it could be used as medicinal and food dual-use only within a limited range and dose (Commission, 2016). The dried fruit spikes are included in the Chinese Pharmacopoeia (2020 Edition) as commonly used Chinese medicinal materials. According to the theory of TCM, the medicinal nature of it is cold, the medicinal flavor of it is bitter and acrid, and enters the foot jueyin liver channel and the foot shaoyang gallbladder channel. It has the effects of clearing the liver and vision-improving, dissipate masses and detumescence, and is used for red swelling and pain of eyes, night pain of eyeballs, headache and vertigo, breast carbuncle, scrofula and hypertension (Commission, 2020). PVL has complex structures and a large number of chemical constituents. Triterpenoids, sterols, flavonoids, phenylpropanoids, organic acids, volatile oils and polysaccharides have been isolated from PVL, and triterpenoids and flavonoids are major active ingredients (Zhang et al., 2018). In addition, the 2020 edition of Chinese Pharmacopoeia stipulated that rosmarinic acid was used as the content determination index of its quality control. Modern pharmacological studies have shown that it has extensive pharmacological activities, and has certain pharmacological effects on anti-tumor, antibacterial and antiviral, anti-inflammatory and immune regulation, antihypertensive, hypoglycemic, hypolipidemic, antioxidant and free radical scavenging, liver protection and so on (Ryu et al., 1992; Fang et al., 2005a; Sárosi and Bernath, 2008; Kim, 2012; Zhang et al., 2014; Huang et al., 2015; Raafat et al., 2016; Çetin and Kalyoncu, 2018; Ahmad et al., 2020; Zhang et al., 2020). More than any of these, its excellent quality and remarkable curative effect have also been highlighted in clinical application. Modern clinical practice is mostly used for the treatment of cancer, hypertension, diabetes, pelvic inflammation, breast hyperplasia, thyroid diseases, and prostate diseases (Li, 2008; Hu and Jiang, 2014; Cheng and Liu, 2017; Lin and Xiong, 2018; Wu, 2018; Nojiri, 2019; Tang et al., 2020b).

Nevertheless, the current research on PVL remains still insufficient, and there are certain limitations in some specific studies. Therefore, in this paper, the botany, ethnopharmacology, traditional applications, phytochemistry, analysis methods, quality control, pharmacological effects and toxicity of it in recent years are comprehensively, deeply and systematically described, and the progress of its research work is also introduced to the readers, so that readers, especially those engaged in the research work in this field, could benefit from it. At the same time, it is also hoped to provide preliminary evidence for future research on PVL, and provide reference for further research on the biological activity and clinical application of TCMs.

## Botany

PVL, originated in Europe and Asia, could be found in temperate regions of the world. PVL is a low, creeping, non-aromatic perennial that is 5–30 cm tall with square stems. Leaves are in opposite pairs along the stem, and are approx 2.5 cm long and 1.5 cm wide. Leaves are oval to lanceolate, untoothed or serrated. Petioles are generally short but can be up to 5 cm in lower leaves. The inflorescence is a dense, oblong or square, whirled cluster with a pair of stalkless leaves below. There are three flowers per bract, and the bracts and calyx are purplish. Flowers are violet (rarely white or pink) and are two-lipped and tubular. The top lip is a concave purple hood. The bottom lip may be white, and has three lobes, with a larger fringed middle lobe. The corolla is 10–14 cm long. It usually blooms from April to June and results from July to October. Seeds are smooth, shiny, brown nutlets that are obovate, oblong, with a convex dorsal side and a roof-like ventral side (Compendium, 2016). In China, there are both wild and artificial cultivation of PVL. The main producing areas are Shaanxi, Gansu, Xinjiang, Henan, Hubei, Hunan, Jiangxi, Zhejiang, Fujian, Taiwan, and other places. They grow in sparse forests, economic forests, barren mountains, ridges, roadsides, ditches, beaches and lakes (Yang and Du, 2016). It enjoys a warm and humid environment. It has strong cold resistance and adaptability, and can grow normally no matter in sandy loam with sufficient sunshine and good drainage, or in dry slopes, foothills, grasslands, roadsides, and fields (Liu et al., 2012). In summer, when the ear is brownish red, it can be collected and used as medicine after removing impurities and drying in the sun. Qi is slight, flovor is light, and the quality is better when the ear becomes purple-brown (Commission, 2020).

It is worth noting that the white flower variety *Prunella vulgaris* var. leucantha and the narrow leaf variety *P. vulgaris* var. lanceolata are easily confused with the original variety *Prunella vulgaris* L. The difference between the former and the original variety is that the flowers are white and produced in southern Sichuan. Growing near a stream at an altitude of 750 m. The latter differes from the original variety in leaf entire, lanceolate to oblong lanceolate, 1.5–4 cm long, 6–10 mm wide, glabrous or sparsely pubescent, yielding Yunnan, Sichuan. Born on roadsides, grass slopes, bushes and forest margins, 3,200 m above sea level (iPlant, 2019).

## Ethnopharmacology

Different regions have unique natural conditions and living customs, and long-term practice have formed unique treatment experience for certain diseases. PVL is common in Britain, Europe, Asia and North America, but its traditional use is generally limited to Asia as a TCM for liver function, goiter and inflammation (Wagner et al., 2020). In 17th century European traditional medicine, the plant is called self-healing and used as a remedy for relieving sore throat, reducing fever and accelerating wound healing (Adamkova et al., 2004). PVL is used in Turkish folk medicine for rheumatism, colds and heart disease (Yesilada et al., 1993). In Japan, it is distributed all over the country. In its folk medicine, it is often used as a diuretic and a medicine with the flowering fruit spikes (Kojima and Ogura, 1986), In Iranian herbal medicine, it is used to treat sore throat, bleeding, pneumonia, headaches, dysentery, hemorrhoids, diabetes and eye inflammation (Morteza-Semnani et al., 2006).

In China, PVL is called “Xiakucao,” while in other western countries, it is often called “common selfheal,” “heal-all,” or “self-heal” (Risk, 2010). It was recorded for the first time in “Shen Nong’s Classic of the Materia Medica” (Shén Nóng Bĕn Căo Jīng, 神农本草经) (Eastern Han Dynasty, AD 25–220), which stated: “It tasted bitter, acrid and cold. Treatment of scrofula, rat fistula, head sores, break concretions, scattered gall, qi stagnation, swollen feet, and removing dampness to relieve paralysis (Wu, 1996)”. Most later generations followed this statement. Otherwise, we summarized the records of PVL in Chinese ancient herbal works in order to understand its efficacy and use more intuitively and clearly (Supplementary Table S1). Current Chinese Pharmacopoeia (2020 Edition) recorded: “acrid, bitter, cold. It belongs to the liver and gallbladder meridians. It mainly has the effects of clearing liver and purging fire, which could improve vision, dissolve swelling and dissipate masses” (Commission, 2020).

All previous editions of Chinese Pharmacopoeia and most ancient books described it with acrid, bitter, cold, but the property of cold is doubtful. “New Compilation of Materia Medica” (Bĕn Căo Xīn Biān, 本草新编) (Qing Dynasty, AD 1644-1911) recorded: “Taste is bitter, qi is warm and the statement of cold is wrong. It belongs to lung, spleen and heart meridians” (Chen, 1996). Besides, in the book of “Encountering the Sources of the “Classic of Materia Medica”” (Bĕn Jīng Féng Yuán, 本经逢原) (Qing Dynasty, AD 1617–1700), it was described as bitter, warm and nontoxic (Zhang, 1996). Modern scholar Bu et al. also discussed its medicinal property and thought that its nature was warm and its medicinal property rose (Bu and Hu, 2009).

## Traditional Applications

### Herb-Pairs

TCM couplet, also known as “herb-pairs,” refers to the commonly used and relatively fixed compatibility form of TCM in clinic, and is the smallest unit of prescription (Duan et al., 2009). They are widely used in clinic. From a certain point of view, the compatibility and application of drug pairs are of special significance. The application of this method has the effects of increasing efficiency, reducing toxicity, so it could better play the efficacy of TCM and improve the clinical therapeutic effect (Meng et al., 2009). In the ancient book of “Treasury of Words on the Materia Medica” (Bĕn Căo Huì Yán, 本草汇言) (Ming Dynasty, AD 1368-1644), There are the following records: “Treat primary acute mastitis: Equipartitioned *Taraxaci Herba* with PVL, and taken by boiling with wine” (Ni, 2005). Modern studies have shown that this drug pairs can increase interleukin-6 (IL-6) level in breast cancer model mice, inhibit the proliferation and promote the apoptosis of breast cancer cells (Tang et al., 2020a). The couplet medicines of *Pinellia Ternata* and PVL are commonly used in clinical medicine. Ancient and modern physicians had carried out a large number of clinical practices, and found that they had better clinical efficacy for hyperthyroidism, insomnia and depression (Wang et al., 2006; Liu et al., 2009; Liu et al., 2014b).

At present, the clinical application of PVL herb-pairs is also very extensive, but it is mainly guided by the classical theory of TCM. Most of them lack a relatively sophisticated basis for modern pharmacological research. Most studies are not deep enough, and the combination with clinical research is not close enough. Therefore, in the future, the compatibility of PVL should be systematically and deeply studied from the aspects of pharmacodynamic material basis, metabolic process, mechanism of action and clinical compatibility application, Only in this way can we understand it more comprehensively and provide better guidance in its clinical application.

### Prescription

Prescriptions select the appropriate drugs under the guidance of the theory of TCM, in accordance with fomula composing principles. After careful consideration of its dosage and usage, it is prepared through proper compatibility (Zuo and Xu, 2016). In this section, we summarize the various prescriptions in the current Chinese Pharmacopoeia (2020 edition) that use PVL as the main drug and briefly list their compositions and functions (Supplementary Table S2).

PVL mainly enters the liver meridian, so it has significant curative effect on breast diseases such as hyperplasia of mammary glands, acute mastitis, breast cancer and other diseases attributed to the liver meridian. In Thoroughly Revised Materia Medica (Bĕn Căo Cóng Xīn, 本草从新, Tang Dynasty, AD 618–907), it is recored that PVL can treat acute mastitis, breast cancer. According to the Yaozh Database (https://db.yaozh.com), there are many kinds of TCM prescriptions and proprietary Chinese medicine prescriptions for the treatment of hyperplasia of mammary glands. Among them, the TCM prescriptions of Ruheyin and Rukuaixiao Decoction, which take PVL as main drug, have definite curative effect on hyperplasia of mammary glands. There are 44 kinds of proprietary Chinese medicine prescriptions for the treatment of hyperplasia of mammary glands, among these, 22 prescriptions contain PVL. Thus, PVL has a high frequency of medication in the clinical treatment of HMG, and to a certain extent, it also shows its important clinical application value.

At present, PVL oral liquid is commonly used in the treatment of hyperplasia of mammary glands, which can relieve breast pain and conducive to dissipation of breast nodules or masses. More in-depth scientific studies are needed in the future to explore the mechanism of PVL for the treatment of breast diseases, so that innovative drugs with low toxicity, economy and high efficiency can be developed.

### Dietotherapy History

As a medicinal and edible perennial herb, PVL has been used in China for thousands of years. PVL is first found in “Extension of the Materia Medica” (Bĕn Căo Yăn Yì, 本草衍义) (Song Dynasty, AD 960–1279) as an edible history, that is: “When the leaves are new and tender, they can be eaten as a vegetable and must be washed to remove the bitter water” (Kou, 1990), and then “Materia Medica Arranged According to Pattern” (Zhèng Lèi Bĕn Căo, 证类本草) (Song Dynasty, AD 960–1279) cited the above content. “The Grand Compendium of Materia Medica” (Bĕn Căo Gāng Mù, 本草纲目) (Ming Dynasty, AD 1578) also introduced the edible method of it: “After cleaning the tender seedlings, soak them away and mix them with oil and salt for edible” (Li, 2005). “Food as Materia Medica” (Shí Wù Bĕn Căo, 食物本草) (Ming Dynasty, AD 1368–1644) also pointed out: “PVL is bitter, cold, non-toxic. The seedlings are washed, soaked to remove the bitterness, stirred with oil and salt, and eaten as a pickle with a very delicious taste” (Lu, 2015). Thus, as early as more than a thousand years ago, it has been used as food and vegetable for people to widely eat.

Particularly, it could also be used for food therapy, cooking into “PVL and Lean pork soup,” and other medicinal food, could clear liver and improve vision, dispel heat and eliminate stagnation (Cui, 2020). “PVL and *Angelica sinensis* Porridge” can be used as a diet to treat breast hyperplasia with good results (Liu and Zhao, 2018). “PVL and Crucian Soup” could treat thyroid adenoma with satisfactory curative effect. The cold nodules disappeared and all the symptoms were eliminated after taking it more than 2 months (Cheng, 1980). It had been reported that soup made of PVL and turtle was suitable for patients with malignant lymphoma at all stages (Li, 2009).

The climate in Guangdong Province of China is hot and humid. Drinking herbal tea is an important part of the dietary habits of people there (Mei, 2005). It is precisely because of the dietary habits, so it is necessary to put various TCM into tea. As early as the Ming Dynasty, Sheganqiao tea prescription containing PVL has appeared in “Herbal Huiyan” (本草汇言) with the effect of clearing away heat and detoxifying, detumescence and dispersing after consumption. In modern, Wang Laoji, He Qizheng and other herbal teas added it, using its heat-clearing function to achieve the effect of eliminating dampness and heat, preventing heat and reducing temperature.

## Phytochemistry

Phytochemicals are active and healthy substances contained in plants. Known as “the gift of plants to human beings.” Up to now, many phytochemical studies have been carried out on PVL, and seven kinds of chemical components have been isolated and identified from this plant, up to more than 250 compounds. In addition to the chemical constituents found in the spikes, the chemical constituents in other parts of it, including leaves, roots and stems, are also comprehensively reported. In this chapter, the chemical constituents isolated and identified by scholars are classified in tabular form (Supplementary Table S3), and the corresponding structures are introduced in the form of figures. Furthermore, PVL contains abundant trace elements. Higher contents of Cu, Fe and Ni in roots (Zhang et al., 2009), the content of each element in panicle from high to low was: Cu, Zn, Cr, Fe, and Cd (Jiang et al., 2008b), the content of each element in stem from high to low was: Fe, Cu, Zn, Cr, Ni, and Cd (Jiang et al., 2009). Modern medicine has proven that trace elements are closely related to human health, growth and development and disease prevention (Yuan and Kang, 2009).

### Triterpenoids

The terpenoids in PVL are mainly triterpenoids. Triterpenoids are a group of terpenes with a parent nucleus consisting of 30 carbon atoms. They exist in plants in free form or in the form of glycoside combined with sugar or in ester form. There are three main types of triterpenoids in PVL, including oleanane, ursane and lupane, respectively. Triterpenoids are the major active ingredients of PVL and its pharmacological activities such as anti-inflammatory, antibacterial, liver protection, anti-diabetes, anti-cancer, and anti-virus (Feng et al., 2011a; Jie et al., 2013; Zhou et al., 2013; Zhang et al., 2015; Pan, 2018b; Li et al., 2019a). In this part, a total of 87 triterpenoid constituents are isolated and identified, the chemical stractures of triterpenoids are particularly shown in Tables 1–6.

**TABLE 1:** Chemical structures of triterpenes isolated from PVL─Part I.

	Groups	R_1_	R_2_	R_3_	R_4_	R_5_	R_6_
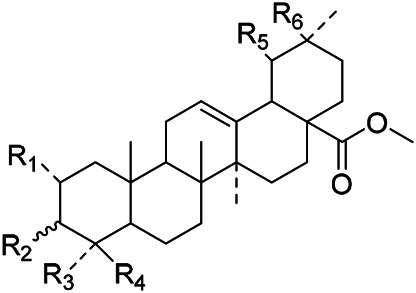	(26) Methyl ursolate	H	βOH	Me	Me	Me	H
	(28) Methyl oleanolate	H	βOH	Me	Me	H	Me
	(73) Methyl 2α,3α-dihydroxyurs-12-en-28-oate	OH	αOH	Me	Me	Me	H
	(32) Methyl 3-epimaslinate	OH	αOH	Me	Me	H	Me
	(75) Methyl 2*α*,3*α*,24-trihydroxyursa-12,20 (30)-dien-28-oate	OH	αOH	Me	CH_2_OH	Me	H
	(33) Methyl 2α-hydroxyursolate	OH	βOH	Me	Me	Me	H
	(29) Methyl maslinate	OH	βOH	Me	Me	H	Me
	(76) Methyl 2α,3α,23-trihydroxyolean-12-en-28-oate	OH	αOH	CH_2_OH	Me	H	Me
(Kojima and Ogura)	(77) Methyl 2α,3α,24-trihydroxyolean-12-en-28-oate	OH	αOH	Me	CH_2_OH	H	Me

**TABLE 2:** Chemical structures of triterpenes isolated from PVL─Part II.

	Groups	R_1_	R_2_	R_3_	R_4_
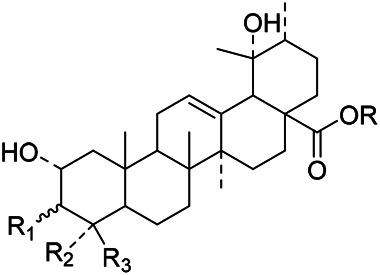	(22) Pruvuloside A	αOH	CH_3_	CH_3_	Glc2-Glc
	(19) Niga-ichigoside F1	βOH	CH_2_OH	CH_3_	Glc
	(20) Niga-ichigoside F2	αOH	CH_2_OH	CH_3_	Glc
(Zhang and Yang)	(23) Pruvuloside B	αOH	CH_3_	CH_2_OH	Glc

**TABLE 3:** Chemical structures of triterpenes isolated from PVL─Part III.

	Groups	R_1_	R_2_	R_3_
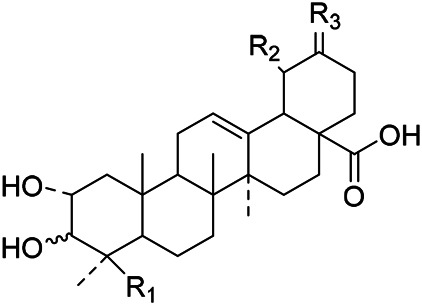	(45) 2*α*,3*α*,24-trihydroxyursa-12,20 (30)-dien-28-oic acid	CH_2_OH	CH_3_	CH_2_
	(49) 2*α*,3*α*,24-trihydroxyolean-12-en-28oic acid	CH_2_OH	H	CH_3_CH_2_CH_3_
	(51) 2*α*,3*α*,24-trihydroxy-12-en-28-ursolic acid	CH_2_OH	CH_3_	CH_3_CH_3_
	(61) 2*α*,3β-dihydroxy-12-en-28-ursolic acid	H	H	CH_3_CH_2_CH_3_
(Wang et al.)	(62) 2*α*,3β-dihydroxy-12-en-28-oleanolic acid)	H	CH_3_	CH_3_CH_3_

**TABLE 4:** Chemical structures of triterpenes isolated from PVL─Part IV.

	Groups	R_1_	R_2_	R_3_	R_4_	R_5_	R_6_	R_7_	R_8_	R_9_
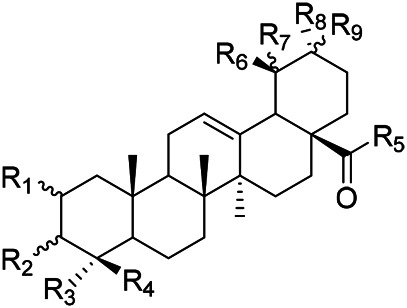	(3) Maslinic acid	αOH	αOH	CH_3_	CH_3_	H	CH_3_	αOH	CH_3_	H
	(48) 2*α*,3*α*,23-trihydroxy-12-en-28-ursolic acid	αOH	βOH	CH_3_	CH_3_	H	H	H	CH_3_	CH_3_
	(54) 2*α*,3*α*,19*α*-trihydroxyurs-12-en-28-oic acid	αOH	αOH	CH_2_OH	CH_3_	H	CH_3_	αOH	CH_3_	H
	(57) 2*α*,3*α*,19*α*,23-tetrahydroxyurs-12en-28oic acid	αOH	αOH	CH_2_OH	CH_3_	H	CH_3_	H	CH_3_	H
	(59) 2*α*,3*α*,19*α*,24-tetrahydroxyurs-12en-28oic acid 28-*O*-*D*-glucopyranoside	αOH	βOH	CH_2_OH	CH_3_	Glc	CH_3_	αOH	CH_3_	H
(Lee et al.)	(69) 2*α*,3*β*,19*α*,24-tetrahydroxyurs-12en-28oic acid 28-*O*-*D*-glucopyranoside	αOH	αOH	CH_2_OH	CH_3_	Glc	CH_3_	αOH	CH_3_	H

**TABLE 5:** Chemical structures of triterpenes isolated from PVL─Part V.

	Groups	R_1_	R_2_	R_3_	R_4_	R_5_	R_6_	R_7_	R_8_	R_9_
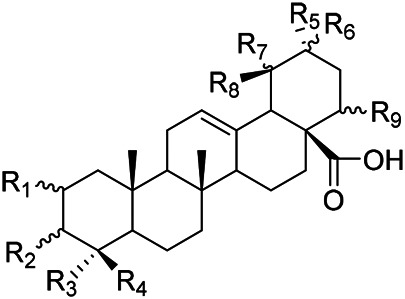	(84) 2,3,24-trihydroxyolean-12-en-28-oic acid	OH	OH	CH_3_	CH_2_OH	CH_3_	CH_3_	H	H	H
	(83) 2,3,24-trihydroxyurs-12-en-28-oic acid	OH	OH	CH_3_	CH_2_OH	CH_3_	H	H	CH_3_	H
	(42) 3β,22α-dihydroxyolean-12-en-28-oic acid	H	βOH	CH_3_	CH_3_	CH_3_	CH_3_	H	H	αOH
	(43) 3β,22α-dihydroxyurs-12-en-28-oic acid	H	βOH	CH_3_	CH_3_	CH_3_	H	H	CH_3_	αOH
(Yang et al.)	(87) 22-hydroxy-3-oxoleana-12-en-28-oic acid	H	=O	CH_3_	CH_3_	CH_3_	CH_3_	H	H	H

**TABLE 6:** Chemical structures of triterpenes isolated from PVL─Part VI.

	Groups	R_1_	R_2_	R_3_	R_4_	R_5_	R_6_
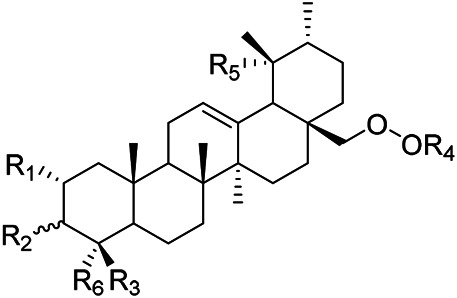	(52) 2α,3α-24-trihydroxyursa-12-en-28-oic acid-28-O-β-D-glucopyranoside	OH	αOH	CH_2_OH	Glc	H	CH_3_
	(70) 2α,3β,19α,24-tetrahydroxyurs-12-en-28-oic acid-28-β-D-glucopyranoside	OH	βOH	CH_2_OH	Glc	OH	CH_3_
	(55) 2α,3α,19α-trihydroxyurs-12-en-28-oic acid-28-β-D-glucopyranoside	OH	αOH	CH_3_	Glc	OH	CH_3_
	(56) 2α,3α,19α-trihydroxyurs-12-en-28-oic acid-28-β-D-glucopyranosyl-(1→2)-β-D-glucopyranoside	OH	αOH	CH_3_	Glc (1–2)Glc	OH	CH_3_
(Yu et al.)	(82) Rotundic acid 28-O-α-D-glueopyranosyl (1→6)-β-D-glueopyranoside	H	βOH	CH_3_	Glc (1–6)Glc	OH	OH

Oleanolic acid (1), Ursolic acid (2) and *β*-Amyrin (4, isolated from this genus for the first time) were isolated from PVL, and they proved that (1) and (2) and their saponines both have anti-hypertensive activity, and the saponines have anti-cardiac arrhythmic activity (He et al., 1985). Japanese scholar Kojima et al. isolated a new triterpene, methyl 2*α*,3*α*,24-trihydroxyolean-12-en-28-oate (76) (Kojima and Ogura, 1986). Subsequently, three new pentacyclic triterpenoids (73, 74, 80) and two new hexacyclic triterpenoids (78, 79) were isolated as their methyl esters (Kojima et al., 1987; Kojima et al., 1988). Zhang et al. isolated two new ursane triterpenoid saponins pruvuloside A and B (22, 23) (Zhang and Yang, 1995). Two new compounds, vulgarsaponin A and B (24, 25), were isolated from the dried fruitspikes of PVL (Wang et al., 1999; Tian et al., 2000). Similarly, a new triterpenoid saponin established to be prunelloside A (21) was isolated (Zhang et al., 2008). South Korean scholar Byun et al. first isolated euscaphic acid (6) and 3*β*-Hydroxyolean-5,12-diene (39) from the spikes of PVL. Furthermore, they proved that (39) exhibited significant cytotoxic activity against human colon adenoblastoma (HT-29) (Byun et al., 2007). Korean scholar Lee et al. isolated and identified (3), (54), (57), (58), (69), and proved ursolic acid exhibited moderate cytotoxic activity against A549, SK-OV-3, SK-MEL-2, and HCT15 cells (Lee et al., 2008). Du et al. isolated a new, unusual ^∆11(12)^ triterpene, 3*β*,13*β*-dihydroxyolic-11-ene-28-oic acid (44) with anti-complementary activity against the classical pathway and the alternative pathway (Du et al., 2012). Yu et al. investigated the methanol extract from the spikes of it, and led to the isolation of two new pentacyclic triterpenoid glycosides Vulgasides I (17) and II (18) (Yu et al., 2015).

### Sterols

Sterols are steroids containing hydroxyl groups. They are all based on cyclopentane phenanthrene. The Sterols in PVL are mostly phytosterols and their saponins, such as sitosterol and stigmasterol. Daucosterol (90) was first isolated from the dichloromethane extract of PVL (He et al., 1985). Kojima et al. obtained *α*-spinasterol (91) and stigmast-7-enol (95) from the stems and leaves of PVL (Kojima and Ogura, 1986). Meng et al. isolated *β*-sitosterol (93), (22E,20S,24S)-stigmasta-7,22-diene-3-one (100), in which compound (100) was obtained from this genus for the first time (Meng and He, 1995). Tian et al.first isolated stigmasterol (88) and stigmast-7-en-3*β*-ol (96) (Tian et al., 2000). Lou et al. isolated a new C^21^ steroidal glycoside, named qingyangshengenin-3-*O*-*β*-*D*-digitoxopyranoside (97) and a known steroidal glycoside qinyangshengenin-3-*O*-*β*-*D*-oleandropyranosyl-(1→4)-*β*-D-cymaropyranosyl-(1→4)-*β*-*D*-digitoxopyranoside (98) (Lou et al., 2015). South Korean scholar Choi et al. isolated spinasterone (89), 5-stigmasta-7, 22-dien-3-ol (99) (Choi et al., 2016). The corresponding chemical structures are shown in Table 7.

**TABLE 7:** Chemical structures of sterols isolated from PVL.

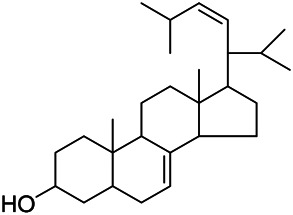	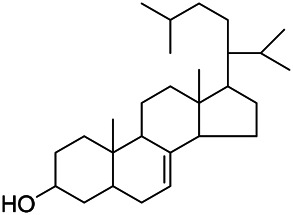	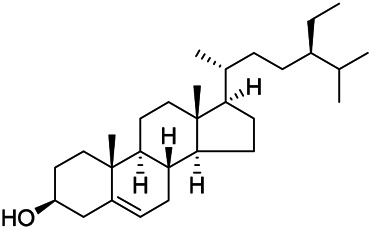
(95) Stigmast-7-enol	(91) *α*-Spinasterol	(89) Spinasterone
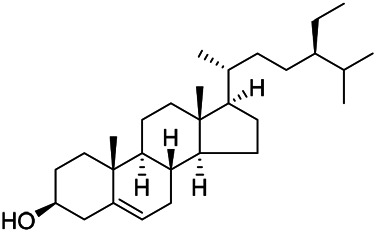	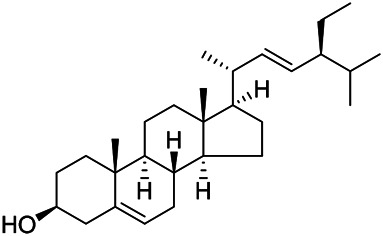	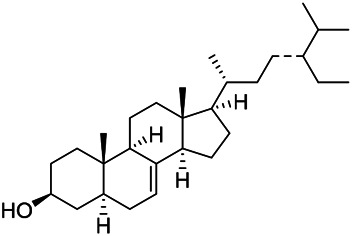
(93) *β*-Sitosterol	(88) Stigmasterol	(96) Stigmast-7-en-3*β*-ol
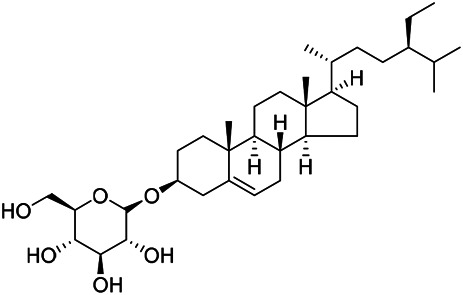	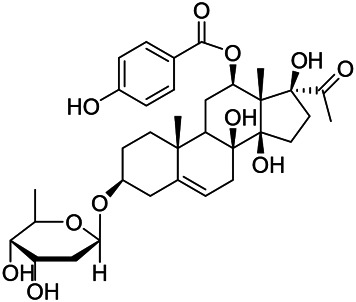	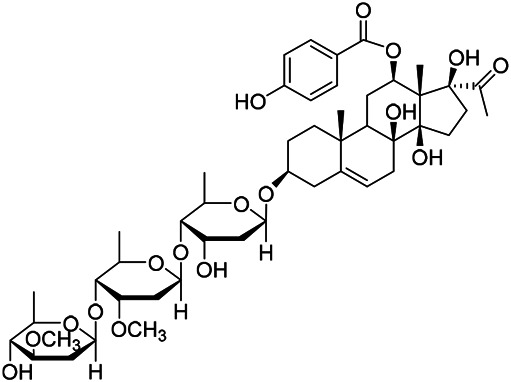
(90) Daucosterol	(97) Qingyangshengenin-3-*O*-*β*-*D*-digitoxopyranoside	(98) Qinyangshengenin-3-*O*-*β*-*D*-oleandropyranosyl-(1→4)-*β*-*D*-cymaropyranosyl-(1→4)-*β*-*D-*digitoxopyranoside

### Flavonoids

Flavonoids are a series of compounds composed of two phenolic hydroxyl benzene rings (A and B rings) connected by central tricarbon atoms. The basic nucleus of flavonoids is 2-phenylchromone. The flavonoids in PVL can be divided into flavone, flavanonole, and flavanone according to their chemical structures. The isolated compounds and their corresponding structures are shown in Table 8. PVL total flavonoids have a wide range of biological and pharmacological activities, such as anti-tumor, anti-oxidation, free radical scavenging, anti-inflammation, inhibiting osteoporosis, and improving osteoarthritis (Yan et al., 2012; Liu et al., 2014a; Li et al., 2019c; Shao et al., 2019; Song et al., 2020; Lu et al., 2022).

**TABLE 8:** Chemical structures of flavonoids isolated from PVL.

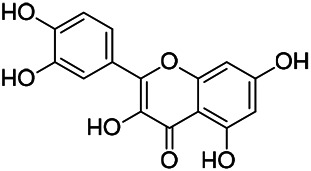	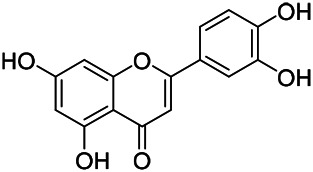	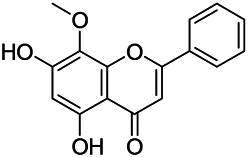
(132) Quercetin	(134) Luteolin	(136) Wogonin
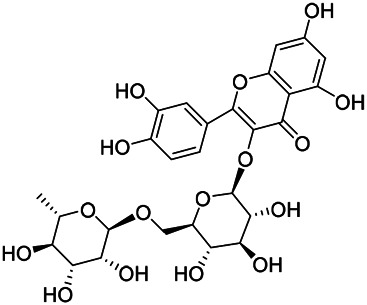	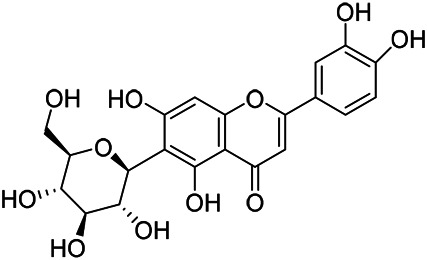	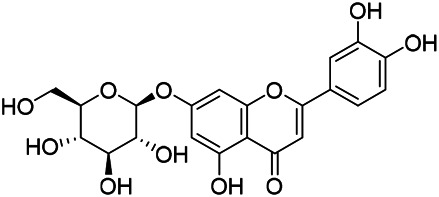
(130) Rutin	(137) Homoorientin	(135) Cynaroside
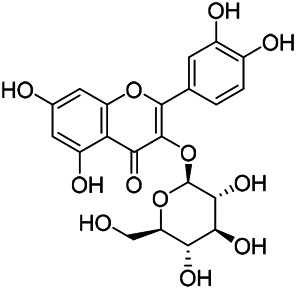	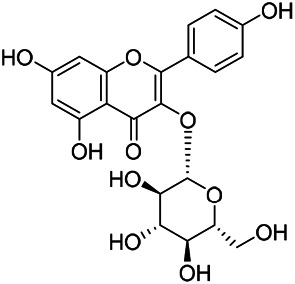	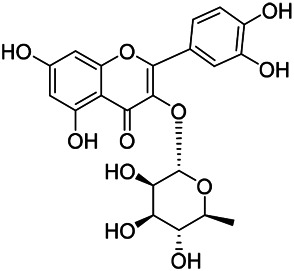
(143 ) Quercetin-3-*O*-glucoside	(145) Kaempferol-3-*O*-glucoside	(133) Quercitrin
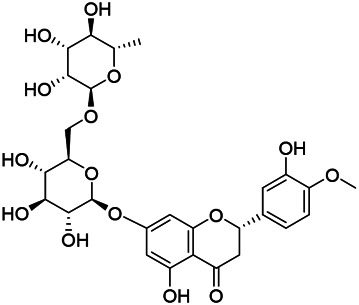		
(138) Hesperidin		

Dmitruk et al., (1987) isolated luteolin (134), homoorientin (137) and cynaroside (135) from the ethanol extract of the aboveground part of it, in which compound (134) was first isolated. Zhang et al. isolated quercetin-3-*O*-glucoside (143), kaempferol-3-*O*-glucoside (145) from the methanol extract of it from France (Zhang and Yang, 1995). Wang et al. isolated rutin (130), quercetin (132), hesperidin (138), quercetin-3-*O*-*β*-*D*-galactoside (140) and quercetin-3-*O*-*β*-*D*- glucoside (141) from dried ears. Among them, compound (138) and (141) were isolated from this genus for the first time (Wang et al., 1999; Wang et al., 2008). Subsequently, Gai et al. isolated kaempferol-3-*O*-*β*-*D*-glucoside (146) from 70% ethanol extract of it (Gai et al., 2010). Zhang et al. identified acacetin-7-*O*-*β*-*D*-glucopyranoside (149) from the dried ear, which was isolated from this genus for the first time (Zhang et al., 2008). Korean scholar Lee et al. isolated from the aerial part quercetin-3-*O*-*β*-*D*-glucopyranoside (142), kaempferol-3-*O*-*β*-*D*-glucopyranoside (147), quercertin-3-*O*-*α*-*L*-rhamnopyranosyl (1→6)-*β*-*D*-glucopranoside (144), kaempferol 3-*O*-*α*-*L*-rhamnopyranosyl (1→6)-*β*-D-glucopranoside (148) (Lee et al., 2008). And then, the following compounds wogonin (136), quercitrin (133) were isolated and identified by different scholars (Gai et al., 2010; Yu et al., 2012).

### Phenylpropanoids

Phenylpropanoids are naturally occurring compound consisting of a class of benzene rings connected with three straight-chain carbons (C6-C3 groups). Phenylpropanoids in PVL are mainly phenylpropionic acids and coumarins, and their corresponding structures are shown in Table 9. The active ingredient rosmarinic acid in PVL has been proved to have a variety of pharmacological effects, such as anti-invasion, anti-oxidant, anti-inflammatory and immune regulation (Zdařilová et al., 2009; Xu et al., 2010b; Djivanovna et al., 2018). Dmitruk et al. isolated three coumarin compounds from the ethanol extract of the epigeal parts of it: umbelliferone (107), scopoletin (106), and esculetin (108) (Dmitruk, 1986). Wang et al. isolated ethyl caffeate (103, for the first time from this genus), rosmarinic acid (110) and its derivatives methyl rosmarinate (111), ethyl rosmarinate (113), butyl rosmarinate (114). Meanwhile, 3, 4, *α*-trihydroxy-butyl phenylpropionate (126) and 3,4, α-trihydroxy-methyl phenylpropionate (127) were isolated from this genus (Wang et al., 1999; Wang et al., 2000; Wang et al., 2001). Yan et al. isolated and identified caffeic acid-3-*O*-glucoside (104), *trans*-salviaflaside methyl ester (118), *trans*-salviaflaside (119), (-)-syringaresinol-4-*O-β-D*-glucopyranoside (128) (Yan et al., 2016). Gu et al. isolated Danshensu (120) from the spikes of it for the first time. Then Gai et al. isolated methyl 3,4-dihydroxyphenyl lactate (121) and ethyl 3,4-dihydroxyphenyl lactate (122) from 70% ethanol extract (Gu et al., 2007; Gai et al., 2010). Later, researchers have successively isolated and identified different chemical constituents such as: caffeic acid (101), caffeic acid-*O*-hexoside (105), *p*-hydroxycinnamic acid (124) and so on (Tian et al., 2000; Lee et al., 2008; Yang et al., 2016).

**TABLE 9:** Chemical structures of phenylpropanoids isolated from PVL.

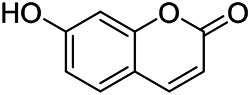	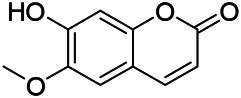	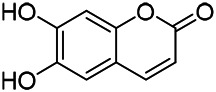
(107) Umbelliferone	(106) Scopoletin	(108) Esculetin
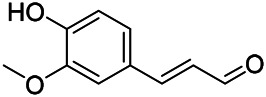	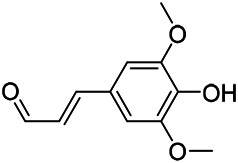	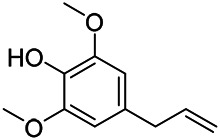
(109) Coniferaldehyde	(117) Sinapaldehyde	(123) 4-allyl-2,6-dimethoxyphenol
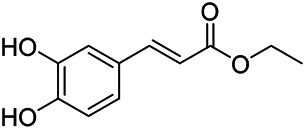	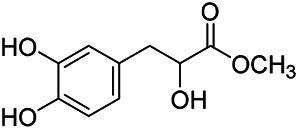	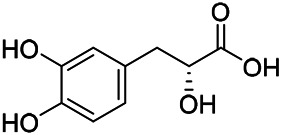
(103) Ethyl caffeate	(126) 3,4, *α*-trihydroxy-butyl phenylpropionate	(120) Danshensu
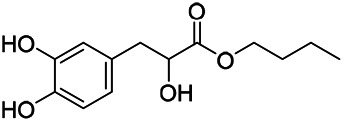	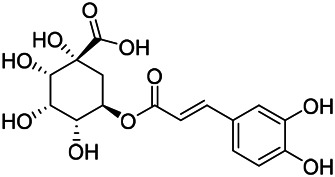	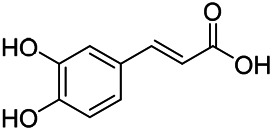
(127) 3,4, α-trihydroxy-methyl phenylpropionate	(102) Chlorogenic acid	(101) Caffeic acid
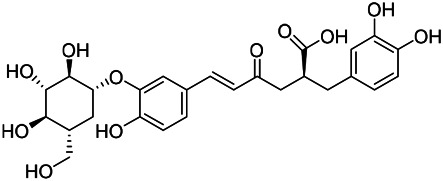	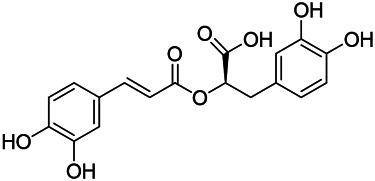	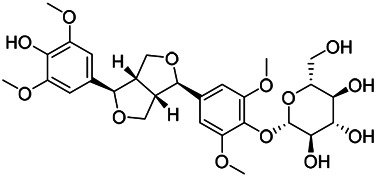
(116) Salviaflaside	(112) Rosmarinic acid	(128) (-)-syringaresinol-4-*O-β-D*-glucopyranoside
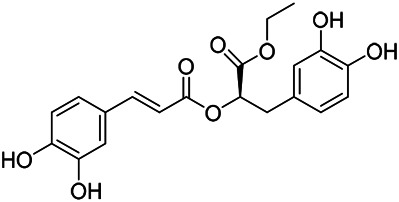	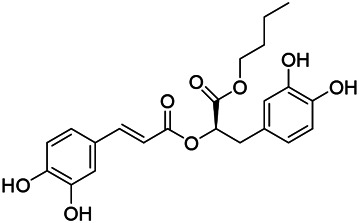	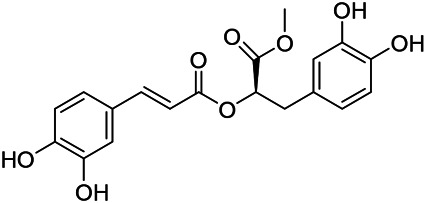
(115) Ethyl rosmarinate	(114) Butyl rosmarinate	(113) Methyl rosmarinate

### Volatile Oils

Volatile oils, also known as essential oils, are aromatic, volatile liquids obtained from plant material through steam distillation and named after the plant from which they are derived. The volatile oil of PVL mainly includes terpenes (monoterpenes, sesquiterpenes and their oxygenated derivatives), small molecule aliphatic compounds and aromatic compounds.

A large number of pharmacological experiments have shown that volatile oil has pharmacological activities such as anti-inflammatory, antioxidant, antifungal, antitumor, and has a variety of applications in medicine (Lei, 2019; Lawson et al., 2020; Mak and Walsh, 2021). Yang et al.used GC/FT-IR technology to analyze the components of the essential oil of it, and the main components were identified as 1,8-cineol (223) (44.827%), *β*-pinene (192) (15.736%), linalyl acetate (213) (4.187%), *α*-phellandrene (189) (5.578%) (Yang et al., 1988). Wang et al. used GC-MS to analyze the components of volatile oil from the spikes of domestic PVL, and identified 12 compounds (224, 227, 234, 235, and 243–250) (Wang et al., 1994). Iranian scholar Morteza et al. extracted volatile oil from the aerial part of dried flowering spikes, and identified 32 components. The main components were Selin-11-en-4-*α-*ol (237) (14.9%), *cis*-eudesma-6,11-diene (220) (9.4%), 1,10-di-*epi*-cubenol (222) (8.0%), spathulenol (184) (5.8%) and Geracrene D (183) (5.1%) (Morteza-Semnani et al., 2006). Yang et al. carried out qualitative and quantitative analysis of volatile components in different parts of it, and the result showed that 145 components were identified, of which compound (-)-Thujopsen (182), *α*-Calacorene (188), Sinapaldehyde (202), Ferruginol (204) and some other volatile oils were found in PVL (Yang and Zhao, 2019). Chemical structures of volatile oils isolated from PVL were shown in Table 10.

**TABLE 10:** Chemical structures of volatile oils isolated from PVL.

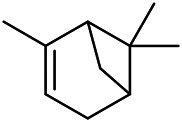	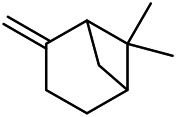	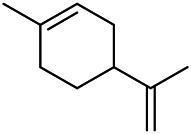	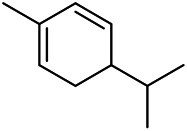	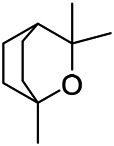
(191) *α*-Pinene	(192) *β*-Pinene	(199) *D*-Limonene	(189) *α*-Phellandrene	(223) 1,8-Cineol
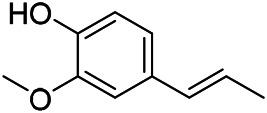	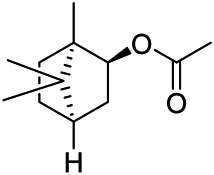	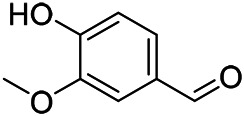	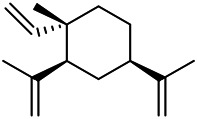	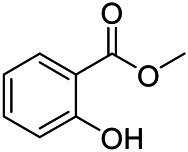
(205) *cis*-Isoeugenol	(216) Bornyl acetate	(200) Vanillin	(197) *β*-Elemene	(215) Methyl salicylate
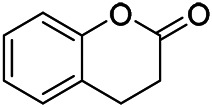	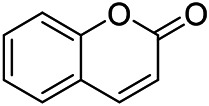	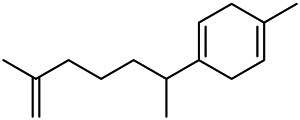	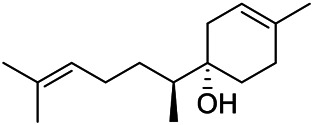	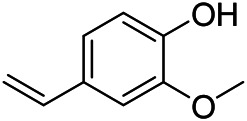
(111) 3,4-Dihydrocoumarin	(110) Coumarin	(194) *β*-Curcumene	(193) *β*-Bisabolol	(206) 4-Vinylguaiacol
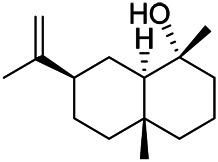	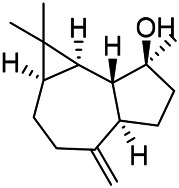	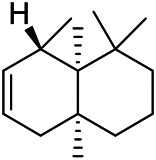	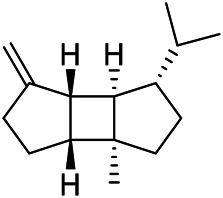	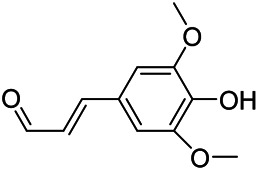
(237) Selin-11-en-4-*α-*ol	(184) Spathulenol	(182) (-)-Thujopsen	(196) *β*-Bourbonene	(202) Sinapaldehyde
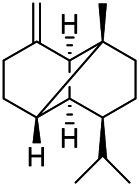	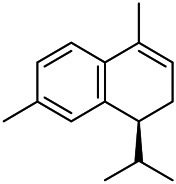	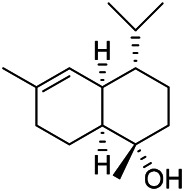	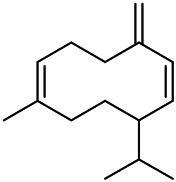	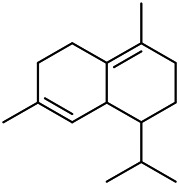
(195) *β*-Copaene	(188) *α*-Calacorene	(190) *α*-Muurolol	(183) Germacrene D	(198) *δ*-Amorphene
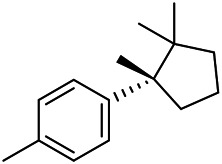	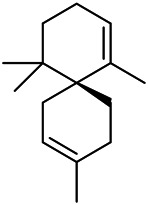	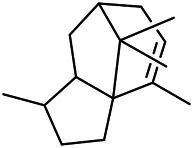	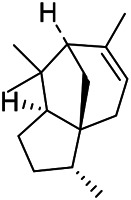	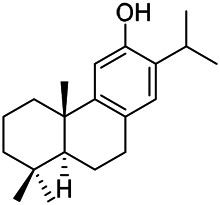
(181) Cuparene	(188) *α*-Chamigrene	(185) *α*-Patchoulene	(186) *α*-Cedrene	(204) Ferruginol

### Organic Acids

Organic acids are widely distributed in leaves, roots, especially in fruits. Fatty acids are common organic acids in PVL, such as linoleic acid (171), linolenic acid (170) and arachidic acid (165), which are essential fatty acids for human body. Besides, there also exists malonic acid (157), pentadecanoic acid (160), palmitoleic acid (167) (Tian et al., 2000; Yang and Zhao, 2019). Aromatic organic acids such as benzoic acid (154), 4-Hydroxybenzoic acid (169) (Tian et al., 2000; Zheng, 2012; Yang and Zhao, 2019). Except for a few free fatty acids, Fatty acids are mostly esterified with glycerol or waxed with higher alcohols such as palmitic acid ethyl ester (162), methyl linolenate (173), monopalmitin (168) (Yang and Zhao, 2019). Some organic acids are the components of resin such as dehydroabietic acid (172) (Yang and Zhao, 2019). Tian et al. isolated and identified four fatty acid compounds from the methylation of lipophilic part of it, which were stearic acid (163), oleic acid (164), palmitic acid (161), behenic acid (166) (Tian et al., 2000). A new phenolic glycosides, gentisic acid 5-*O*-*β*-*D*-(6′-salicylyl)-glucopyranoside (179), was isolated for the first time by Gu et al. (Gu et al., 2011). Zheng et al. isolated anti-tuberculosis active components methyl 3,4-dihydroxybenzoate 178) (Zheng, 2012). Bai et al. isolated cyclopentaneacetic acid 158) from the spikes of it (Bai et al., 2015). Subsequently, citric acid (150), gluconic acid (152), protocatechuic acid 153) and malic acid 156) were isolated from water extract of PVL by Liang et al., and the contents were 14.8%, 8.2%, 1.8% and 3.9%, respectively (Liang et al., 2013).

### Polysaccharides

Polysaccharides are composed of more than 10 monosaccharides linked by glycosidic bonds, widely distributed in nature. Polysaccharides is one of important active components in PVL, Domestic and foreign scholars have done a lot of work on the extraction, purification and structural analysis of it, and have confirmed its antioxidant and scavenging free radicals, immune regulatory activity, antiviral and antitumor (Xu et al., 1999; Fang et al., 2005b; Xiong and Li, 2010; Hao et al., 2020).

Jordanian scholar Tebba et al. isolated a sulfated polysaccharide (prunellin) with anti-HIV activity from the aqueous extract, with a molecular weight of about 10 kDa. And proved to us that its monosaccharide composition was glucose, galactose, xylose, gluconic acid, galactose acid and galactose amine (Tabba et al., 1989). Zhang isolated and purified the polysaccharide PP2 and confirmed that it was composed of rhamnose, arabinose, mannose, glucose, galactose and other monosaccharides (Zhang, 2006). A new heterpolysaccharide exhibited stable immune activities, meanwhile, called PVL-P1 (1,750 kDa), was isolated from the fruit clusters of PVL, and consisted of arabinose, xylose, mannose, glucose, and galactose with molar percentages of 28.37, 54.67, 5.61, 5.46, and 5.89%, respectively. The main linkage types of PVL-P1 were proved to be (1→5)-linked α-L-Ara, (1→)-linked α-L-Ara, (1→3)-linked α-D-xyl, (1→3)-linked β-D-Gal, (1→3,6)-linked β-D-Gal, (1→3,6)-linked α-D-Man and (1→6)-linked α-D-Glc (Li et al., 2015).

Not long after that, a sulphated polysaccharide PSP-2B (32 kDa) with a sulfate content of 10.59% was isolated from aqueous extracts of it, the major sugars comprising PSP-2B are arabinose, galactose and mannose, in addition to small amounts of glucose and uronic acids. The framework of PSP-2B is speculated to be a branched arabinogalactomannan, and the side chains are terminated primarily by the Araf residues. Moreover, its activity against HSV was demonstrated by a series of experiments (Ma et al., 2016). Subsequently, Du et al. isolated two branched acidic polysaccharides (PW-PS1 and PW-PS2) with anticomplement activity. PW-PS1 was composed of Ara, Xyl, and 4-methoxy-Glc A in a ratio of 1.0:2.6:0.8, and the main linkages of the sugar residues of PW-PS1 included terminal *β*-*D*-Xylp, 1,4-linked *β*-*D*-Xylp, 1,3-linked *α*-*D*-Arap, 1,3,5-linked *α*-*D*-Arap, and terminal 4-methoxy-α-D-Glcp A. PW-PS2 was composed of Rha, Ara, Xyl, Gal, and Gal A in a ratio of 0.6:1.0:1.3:1.8:3.4, and the main linkages between the sugar residues of PW-PS2 included terminal Araf, 1,4-linked β-D-Xylp, 1,3-linked α-D-Rhap, terminal α-D-Galp, and 1,4,6-linked α-D-Galp (Du et al., 2016).

### Others

Except for the seven chemical components described above, many other chemical constituents have been found in PVL, such as autantiamide acetate belonging to oligopeptide, and some quinone compounds: tanshinone I, rhein, chrysophanic acid, 2-hydroxyl-3-methyanraquinone (Gu et al., 2007; Xu et al., 2010a), Of course, in addition to these substances, alkaloids, inorganic salts, vitamins, resins, tannins, proteins and lipids are also present in it.

To date, hundreds of phytochemicals have been isolated and purified from PVL, and their biological activities have also been verified. However, this research mainly focuses on triterpenoids and flavonoids and polysaccharides, while for other chemicals such as phenylpropanoids, organic acids and volatile oil research is still relatively scarce. Therefore, further research is still required. New technologies such as identification, separation and purification of plant chemicals are continuously used to focus on the biological effects and structure-activity relationships of plant chemicals. Actively carrying out scientific and technological research and development in the field of plant chemical substances, giving full play to the advantages of traditional Chinese medicine resources in China, and combining advanced biochemical engineering and genetic engineering technology will be an important breakthrough direction to improve the development level of clinical drugs and health food of PVL.

## Pharmacology

PVL is a commonly used Chinese medicinal material in clinical practice. It has a wide range of pharmacological activities due to its rich chemical composition. In this section, we refer to “General Requirements for developing, conducting and researching medicinal plants and natural products (phytopharmacology)” (Heinrich et al., 2020), the pharmacological activities of PVL in recent years (Supplementary Table S4) are summarized, so as to play a guiding role in the further development and utilization of PVL.

### Anti-Tumor

Early on, Chen et al. carried out a series of cell experiments *in vitro*, found that PVL extract could induce Jurkat cell apoptosis by down-regulating Bcl-2 protein and up-regulating Bax protein, thereby inhibiting the growth of Jurkat human T lymphoma cell (Chen et al., 2009). Some researchers found that the 60% ethanol extract of PVL had a chemopreventive effect on non-small cell lung cancer, and its mechanism of action may be related to promoting apoptosis and regulating cell cycle (Feng et al., 2010a). Thereafter, PVL aqueous extract was found to inhibit the invasion and migration of human liver carcinoma HepG2, Huh-7 and Hep3B cells via attenuating matrix metalloproteinases (Kim et al., 2012). And another thing, especially, the ethyl acetate extract of endophytic fungi from PVL exhibited a growth-suppressive activity on gastric cancer *in vitro* and *in vivo* (Tan et al., 2015).

Not only that, with the exception of extracts, many of the active ingredients from PVL also exhibited significant antitumor activity in recent years. Feng et al. established cell-line-derived xenograft (CDX) mouse models to evaluate the *in vivo* anti-lung adenocarcinoma activity of PVL polysaccharid, and found that it could dramatically increase the thymus index and spleen of tumor-bearing mice index (Feng et al., 2010c). Yang et al. found that hyperoside in PVL could induce apoptosis of human NSCLC A549 cells through mitochondrial apoptotic pathway (Yang et al., 2017). Şahin et al. found that rosmarinic acid in PVL had different degree inhibitory effect on cell proliferation in pancreas (PANC-1), prostate (PC-3), colon (HT-29) and breast (MDAMB 436) and GBM (T98G) cell lines (Şahin et al., 2017). Oleanolic acid from PVL, was proved to induce apoptosis of lung adenocarcinoma cells by down-regulating the expression of Bcl-2 and up-regulating the expression of Bax and Bad (Feng et al., 2011b). In addition, Yang et al. proved through experiments that the protocatechuic aldehyde and caffeic acid derived from PVL exhibited obvious cytotoxicity to Michigan Cancer Foundation-7 (MCF-7) cell (Yang et al., 2020).

### Anti-Viral

As early as 1986, the powerful inhibitory effect of PVL polysaccharide on HIV *in vitro* has been proved by Tabba et al. (Tabba et al., 1989), although its mechanism has not been elucidated. Later, Yao et al. found that the PVL extract inhibited HlV-1 replication in the lymphoid cell line MT-4, monocytoid cell line U937, and peripheral blood mononuclear cells, it antagonized HV-1 infection of susceptible cells by preventing infection attachment to the CD4 receptor (Yao et al., 1992). Subsquently, the *in vitro* and *in vivo* anti-HSV activities of the PVL polysaccharide (PPS-2b) were characterized by Zhang et al. The *in vivo* activities of PPS-2b is demonstrated in two animal infection models, Mechanism studies showed that PPS-2b inactivated HSV-1 directly, blocked HSV-1 binding to Vero cells, and inhibited HSV-1 penetration into Vero cells (Zhang et al., 2007).

Moreover, Ao et al. demonstrated that PVL aqueous extract displayed potent inhibitory effect on SCoV-2 SP (including SPG614 mutant) pseudotyped virus (SCoV-2-SP-PVs) mediated infections, and found that it was able to directly interrupt SCoV2–SP binding to its receptor ACE2 and block the viral entry step (Ao et al., 2021). Similarly, Zhang et al. also demonstrated that PVL aqueous extract displayed a potent inhibitory effect on EBOV-GP pseudotyped virus (EBOV-GP-V)-mediated infection in HUVEC and macrophage cell lines. Mechanism studies showed that anti-Ebola virus activity occurred via binding directly to EBOV-GP pseudotyped virus and blocking the early viral events (Zhang et al., 2016). Li et al. studied anti- Zika virus activity of PVL aqueous extract at animal and cell levels. It was found that it could inhibit the cell lesions caused by Zika virus to a certain extent, inhibit the nucleic acid replication and protein expression of Zika virus, and may play an antiviral role by directly killing Zika virus or inhibiting virus recognition and entry into host cells (Li et al., 2020b). Likewise, Modern pharmacological studies have shown that PVL has significant inhibitory effects on equine infectious anemia virus, infectious hematopoietic necrosis virus, respiratory syncytial virus and influenza A virus apart from the above mentioned viruses (Brindley et al., 2009; Tian et al., 2011; Huang, 2016; Li et al., 2019b). For more information, Supplementary Table S4.

### Anti-Bacterial

The emergence and spread of bacterial resistance have made the infection caused by multidrug-resistant bacteria a serious problem (Jiang S. et al., 2020). *In vitro* experiments showed that PVL had certain inhibitory effects on *Escherichia coli*, *Staphylococcus aureus*, *Bacillus subtilis*, *Penicillium* sp., *Aspergillus niger* and *Pseudomonas aeruginosa* (Wang et al., 2004; Dong et al., 2005; Yang et al., 2013a). Lin et al. took the bacterial vaginitis model of rats and discussed the antibacterial effect of PVL aqueous extract *in vivo* for the first time. The results showed that PVL could significantly resist bacterial vaginitis in rats caused by mixed infection of *Staphylococcus aureus*, *Escherichia coli* and *Pseudomonas aeruginosa* in a dose-dependent manner (Lin et al., 2011). Hu et al. found that the extracts obtained by three different extraction methods of PVL had inhibitory effect on *Staphylococcus aureus*, *Staphylococcus epidermidis* and *Propionibacterium acnes*, and the effect intensity was water extract > ethanol extract > ultrasonic extract. Its antibacterial mechanism is related to the permeability of cell wall and cell membrane (Hu et al., 2015). Interestingly, a related study showed that the total phenol value of water extract was higher, followed by ethanol extract and methanol extract, and the total phenol content was negatively correlated with antibacterial activity. Ethanol extract had the best antibacterial activity against the tested microorganisms (Mahboubi et al., 2015). In addition, some researchers also revealed that triterpenoid saponins of PVL inhibited *Escherichia coli*, and petroleum ether extracts of PVL roots inhibited *Staphylococcus aureus*, *S. pneumonia*, *Enterococcus faecalis* and *K. pneumonia* strains (Pan, 2018a; Dar et al., 2022). Based on the existing research, PVL has shown a variety of antibacterial activities, but the research on it is not deep enough. With the deepening of research, it is hoped that the effective antibacterial components of PVL can be found to lay the foundation for the development of new antibacterial drugs.

### Anti-Oxidant

Many studies have shown that PVL has an obvious antioxidant capacity *in vitro*, which makes it possible to increase its application as a natural antioxidant in the food and drug industries (Lugasi et al., 2006; Sárosi and Bernath, 2008). Zhang et al. found that flavonoids in PVL had obvious antioxidant activity *in vitro* and could significantly scavenge DPPH and OH radicals (Zhang et al., 2011). Song et al. found that different extracts of PVL had certain antioxidant activity. Among them, 70% ethanol extract had relatively high DPPH, OH scavenging activity and lipid peroxidation inhibition activity, aqueous extract had the highest O_2_^−^·scavenging activity (Song et al., 2016). Zhu et al. manifested that the half-scavenging rate (IC_50_) of PVL polyphenols to DPPH radical were 4.59 μg/ml, and that of polyphenols to OH radical were 5.52 μg/ml, indicating that it had strong scavenging free radicals ability (Zhu et al., 2018).

Some related studies have shown that high antiradical, antioxidant (SOD), and low hydrolytic activity resides in the 50% ethanol extract of PVL, and rosmarinic acid, not flavonoids is indicative of the major contribution (Djivanovna et al., 2018). Xia et al. proved that the ethyl acetate fraction of PVL aqueous extract had relatively high content of total flavonoids, total phenolic acids and total triterpenoids, with high scavenging ceapacity of DPPH (3.1 ± 0.38) mmol L^−1^ g^−1^ DW and FRAP (2.56 ± 0.35) mmol L^−1^ g^−1^ DW (Xia et al., 2018b). Tan et al. found that PVL had protective effect on acute restraint stress injury in mice. It could significantly reduce the content of H_2_O_2_, MDA and protein carbonyl in the brain tissue of mice, and enhance the activity of SOD (*p* < 0.05, *p* < 0.01). It further proved that total flavonoids of PVL had the strongest antioxidant capacity, which was 2.79 times of total triterpenoids and 56.7 times of polysaccharides (Tan et al., 2016). Studies have shown that 60% ethanol extract of PVL (P-60) had strong *in vitro* antioxidant activity, and can also significantly inhibit the tumor growth, increasing SOD activity and reducing MDA content in serum of tumor-bearing mice. P-60 contains major active compounds such as caffeic acid, rosmarinic acid, rutin and quercetin. Therefore, it can be speculated that total phenols played an important role in antioxidant activity for inhibition of tumor growth (Feng et al., 2010b).

### Anti-Inflammatory and Immunoregulation

Previous studies have shown that polysaccharide fraction of PVL exhibits both immune stimulatory and anti-inflammatory effects against microbial invasion (Fang et al., 2005b). Zdařilová et al. found that rosmarinic acid (RA) in PVL inhibited up-regulation of IL-1β, IL-6, TNF-α and suppressed expression of iNOS on LPS-induced inflammation in human gingival fibroblasts. They speculated that the effect was presumably linked to anti-inflammatory activitity and use of RA may be relevant in modulating the inflammation process (Zdařilová et al., 2009). Xie et al. found that total triterpenoids from PVL (TTP) could inhibit the secretion of PGE2, TNF-α and IL-6 in LPS-stimulated RAW264.7 cells, and significantly inhibit the gene expression of Jak2 and Stat3. The rusults indicated that TTP had a certain anti-inflammatory effect, and the production of this effect might be related to the Jak/Stat pathway (Xie et al., 2013). Li et al. found that PVL aqueous extract (PVAE) could improve the inflammatory lesions of cornea in allergic conjunctivitis rats, significantly reduce the expression of NLRP3, Caspase-l, and IL-1β inflammatory proteins in corneal tissue, and alleviate the degree of liver edema. Its anti-inflammatory mechanism might be related to NLRP3/Caspase1/IL-1β pathway (Li et al., 2020a). Lin et al. demonstrated *Fallopia Japonica* extract (FJE) and PVL extract (PVE) might have a therapeutic effect on myopia. *In vitro* study manifested FJE + PVE reduced lL-6, IL-8, and TNF-α expression in RPE cells, inhibited inflammation by attenuating the phosphorylation of AKT and NF-κB pathway. Besides, they also inhibited myopia-related TGF-β1, MMP-2, NF-κB expression while increasing type I collagen expression in MFD induced hamster model (Lin et al., 2021). Guo et al. found that PVL had anti-inflammatory and immunoregulatory effects on EAT rats. The thyroid volume, thyroiditis inflammation score and serum thyroglobulin antibody levels of EAT rats were attenuated by PV treatment. Mechanism studies manifested that PVL could attenuates EAT by inhibiting HMGB1/TLR9 Signaling (Guo et al., 2021). For more information, Supplementary Table S4.

### Hypotensive, Hypoglycemic and Hypolipidemic

PVL is a TCM with great application and research value for the treatment of hypertension, hyperglycemia and hyperlipidemia, and its efficacy in these diseases has been verified at the clinical level. Accordingly, many scholars have been attracted to explore and study its pharmacological effects (Ma et al., 2014; Li et al., 2016a; Hong, 2020; Yu et al., 2021). Li et al. found that long-term administration of PVL ethanol extract (PVE) could alleviate the weight loss and polydipsia of diabetic ICR mice. High and low doses of PVE (8, 4 g/kg) could significantly reduce the serum triglyceride, cholesterol, LDL content of diabetic mice and increase HDL content. The results showed that PVE had better hypoglycemic potential (Li et al., 2006). Subsequently, related research found that PVL aqueous extract could inhibit the mRNA expression of α-glucosidase, SGLT-1, GLUT-2 and Na^+^-K^+^-ATPase in Caco-2 cells to delay carbohydrate hydrolysis and influence glucose uptake, thereby, lowering postprandial blood glucose levels (Wu et al., 2010). PVL might prevent development of diabetic atherosclerosis, related experiments showed PVL aqueous extract (PVE) suppressed hyperglycemia and diabetic vascular dysfunction in HFHC diet-db/db mice (Hwang et al., 2012).

Previous studies have shown that total alkaloids of compound PVL could relax rat thoracic aorta in a concentration-dependent manner, and the vasodilation effect was not endothelium-dependent. The mechanism may be related to the inhibition of intracellular calcium release and extracellular calcium influx (Que et al., 2015). Wang et al. found that PVL combined with *Uncaria rhynchophylla* could reduce the systolic blood pressure of spontaneously hypertensive rats and control the myocardial hypertrophy caused by hypertension. Its mechanism was preliminarily believed to be related to the decrease of ANG II, and ET content in serum and the increase of CGRP content in serum (Wang et al., 2017). In order to explore the feasibility of treating obesity with PVL, the effects of PVL on body weight and blood lipid of obese C57BL/6J male mice induced by high sugar high fat diet were studied *in vivo*. It was found that PVL could effectively reduce TG, TC, LDL, and increase HDL (*p* < 0.05) (Li et al., 2016b). Before and after this, some scholars studied the inhibitory activity of PVL on pancreatic lipase, and identified several extracts and monomer compounds with potential antagonistic effects on pancreatic lipase through *in vitro* screening. Zheng et al. proved that when the concentration of PVL methanol extract was 200 μg/ml, the inhibitory effect on pancreatic lipase was 74.7%, the monomer compound quercetin showed good activity (27.4%) at the final concentration of 25 μg/ml, followed by luteolin, and the activity was 17.3% (Zheng et al., 2010). Chen et al. screened four possible pancreatic lipase inhibitors from 80% ethanol extract of PVL, which were caffeic acid, rutin, hesperidin, ursolic acid (Chen et al., 2018). For more information, Supplementary Table S4.

### Hepatoprotective

In the past few decades, studies have shown that complex prescription PVL exhibits a protective effect on acute liver injury induced by CCl_4_ in mice. It could significantly reduce the increase of serum ALT and AST in mice with acute liver injury induced by CCl_4_, and alleviate the damage of CCl_4_ on liver cells (Feng et al., 2011c). Soon afterwards, some scholars found that total triterpenoids from PVL could reduce the activities of ALT and AST in serum of rats with acute liver injury, reduce the level of MDA in liver homogenate, increase the levels of SOD and GSH-Px, and inhibit the expression of CYP2E1 in liver tissue, and its mechanism might be related to the inhibition of lipid peroxidation and CYP2E1 expression (Zhang et al., 2012). Deng et al. found that PVL could significantly improve the metabolic disorder of liver injury induced by alcohol, and could significantly reduce the contents of inflammatory factors (TNF-α, IL-6, IL-1β) and liver function marker enzymes (ALT, AST, and ALP) in serum, which were mainly regulated by phenylalanine, tyrosine, and tryptophan biosynthesis pathways (Deng et al., 2021). Furthermore, related studies have also shown that sulfate polysaccharide from PVL has a protective effect on isoniazid-induced liver injury in mice, and its mechanism might be related to antioxidant stress response and reducing inflammatory response (Wang et al., 2021). Cui et al. demonstrated that total triterpenoids from PVL alleviated fulminant hepatic liver failure in mice by inhibiting over-activated MEK/ERK signaling pathway and inflammatory response (Cui and Ren, 2015). Tian et al. found that PVL could improve the symptoms of autoimmune hepatitis (AIH) mice through anti-inflammatory and anti-apoptotic effects. The results showed that it decreased the expression of pro-inflammatory cytokines IFN-γ and IL-17A, increased the expression of anti-inflammatory cytokines TGF-β, and decreased the expression of BAX and caspase-3 (Tian et al., 2020). For more information, Supplementary Table S4.

### Others

PVL also had multiple effects such as hypnosis, choleretic, antidepressant, anti-renal calculi, improving osteoporosis, and alleviating memory impairment in rats caused by scopolamine (Huang et al., 2013; Liu et al., 2017a; He et al., 2017; Qu et al., 2017; Guo et al., 2020; Xi et al., 2020), whereas, the research on pharmacological effects of PVL is currently imperfect. This is mainly reflected in the following points with possible improvements. First of all, some pharmacological experiments lack a reasonable control, such as whether the positive or negative control is needed. Secondly, only one dose group is set up without the investigation of dose dependence, and it is easy to ignore the minimum effective dose, safe dose and minimum toxicity dose. Thirdly, the number of pharmacological indicators is too little, which is easy to cause the neglect of the potential mechanism of action. Fourthly, only the study of crude substances of TCM is carried out without the comparison of monomer compounds. Finally, In addition to obtaining intuitive index evaluation through animal experiments and clinical experiments, it should also be combined with new technologies and methods of molecular biology, cell biology and histology to further explore its intrinsic activity mechanism. precisely because of these reasons, it is more necessary to conduct in-depth, comprehensive and detailed research and exploration in future until the pharmacological mechanism can be fully clarified, which helps to understand and master the drug effect, so as to better guide the clinical rational use of TCM and lay a clinical foundation for the development of new drugs.

## Analytical Method

In recent years, with the development of drug extraction technology and analysis technology, and taking into account the wide range of pharmacological activities of PVL, the extraction and analysis of effective components of PVL are more and more favored by scientific researchers.

Sample analysis includes sample collection, sample pretreatment, analytical determination and data processing. Sample pretreatment and analytical determination occupy the largest proportion of time distribution in the sample analysis process. The extraction of PVL component analysis has gradually developed from the traditional solvent extraction method with methanol or ethanol as solvent to practical, efficient, environmentally friendly and new technologies, such as supercritical fluid extraction (SFE), ultrasonic extraction (USE), and headspace solid-phase microextraction (SPME) (Jiang et al., 2008a; Yang et al., 2013b). Meanwhile, green and safe solvents such as deep eutectic solvents are also used as solvents for the extraction of components from PVL (Xia et al., 2015; Xia et al., 2018a). In addition, for PVL and most other natural medicinal plants, HPLC is a commonly used analytical method. HPLC is a simple and accurate method, however, it can not provide a comprehensive method for the quality control of drugs (Jiang H. et al., 2020). Therefore, in recent years, with the continuous improvement of analytical methods, it has also developed from a single chromatographic technology to a combined chromatographic technology, accompanied by the emergence of high selectivity and high sensitivity detectors. In this section, we listed several common analytical methods in the component analysis of PVL (Supplementary Table S5).

Most of these analytical methods have the advantages of fast, precise, accurate, high sensitivity, short analysis time, strong separation ability, good selectivity, simple operation, low detection line, and wide application range. For most methods, however, sample pretreatment is complex and time-consuming, equipment is high-end and expensive, reagent consumption is large, which is not conducive to health and environment. It is hoped that with the continuous development of science and technology, some practical, efficient, environmentally friendly, new and cost-appropriate technologies will be widely used in the analysis of TCM components.

## Quality Control

At present, the quality control system of TCM and its preparations in China mainly includes appearance identification, character inspection and content determination of effective components (Zhang, 2013). The quality of TCM is one of the main factors affecting the development of TCM. The material basis for the efficacy of TCM is the effective components of TCM, but the effective components in a single TCM may be dozens or even more. Therefore, the quality control of TCM can be established only by solving the problem of component analysis of complex samples. Chinese Pharmacopoeia provides a legal and scientific reference for drug quality control. For PVL, ursolic acid was selected as the evaluation index in Chinese Pharmacopoeia (2005 edition). Since 2010 edition, rosmarinic acid was used as the evaluation index in Chinese Pharmacopoeia. Therefore, the simple quantitative analysis of single chemical marker (ursolic acid or rosmarinic acid) is not sufficient for the quality control of PVL. It is imperative to establish a method for simultaneous determination of main active components in PVL. In particular, for the purpose of fully understanding the development process of quality control of PVL in recent decades, we listed allegations of effective content determination (Supplementary Table S6).

The quality evaluation system of PVL has developed from single effective component or index component detection system to multi-index and multi-component detection system related to efficacy and toxicity. Most of the fingerprints characterizing the chemical constituents of PVL are HPLC or UPLC fingerprints. Pattern recognition technology is also widely used in analyzing multidimensional fingerprint data of PVL, including principal component analysis (PCA), hierarchical cluster analysis (HCA), grey relational analysis (GRA) and partial least squares discriminant analysis (PLS-DA) (Fang and Lin, 2012; Liu et al., 2017b; Huang et al., 2021). This also provides great help for the quality control, the screening of differential markers, and plant classification of PVL (Pi et al., 2017). Of course, its content determination method also involved infrared spectroscopy (IR) and high performance capillary electrophoresis (HPCE) (Guo et al., 2013; Lu et al., 2016). However, this chemical fingerprint combined with multi-component content determination method has certain limitations. For one thing, fingerprint method focuses on qualitative, large amount of information, but its concept is relatively vague. For another, although multi-index quality evaluation can improve the fuzziness of fingerprint, it is only for a few index components to control the quality of TCM quantitatively. This cannot comprehensively and reasonably evaluate the quality of TCM. Therefore, in the future, the quality of PVL should be comprehensively evaluated with chromatographic fingerprint as the core, including the acquisition of chemical composition information, the correlation study of efficacy and the safety evaluation of PVL. For example, Feng et al. used the combination of bioassay method and HPLC multi-component content determination method to evaluate the quality of PVL more realistically, and truly to control the quality of TCM from the aspects of quantity and effect simultaneously (Feng et al., 2016). This also gives us some enlightenment, only taking into account the above aspects, can we expect to truly achieve the quality of TCM controllable, safe and effective.

## Toxicity

PVL itself is not toxic, this statement has been clearly put forward in the Shennong’s Herbal Classic thousands of years ago. But for medicinal materials, there is no absolute saying. Even poisonous medicinal materials, as long as symptomatic, medication is correct, could become a good medicine. There is no poisonous medicinal material in itself. If used improperly, there will be a series of adverse reactions. According to Chinese Pharmacopoeia, the medicinal dosage of PVL is generally 9–15 g. Because of the bitter and cold nature of it, excessive use may stimulate the gastrointestinal tract, causing diarrhea, abdominal pain and other discomfort. Therefore, it is forbidden for people with deficient cold of spleen stomach. So far, reports of adverse reactions to PVL are rare, and one case of contact dermatitis caused by PVL has been reported (Hu and Xia, 2008). In the long run, the clinical detection of adverse reactions of PVL should be further strengthened in order to improve its safety and better expand its clinical application.

## Conclusion and Prospect

At home and abroad, the relevant research on PVL emerged in endlessly. Therefore, this paper summarized the research status of phytochemistry, analytical methods, quality control, pharmacological effects and toxicity of PVL in recent decades, hoping to bring some help to readers, especially those engaged in this field. At the same time, it is also hoped to provide preliminary evidence for future research on PVL, and provide reference for further research on the biological activity and clinical application of TCM.

First, PVL contains complex and diverse chemical constituents, with triterpenoids, sterols, flavonoids, phenylpropanoids, volatile oils, organic acids and polysaccharides as the main types. So far, more than 250 compounds have been isolated and identified. However, although many chemical components were isolated and identified, the biological activity of only a few components was verified. For most components, especially polysaccharides, there is still a lack of understanding of the mechanism of their physiological activities. The determination of their advanced structure, monosaccharide composition and content remains to be further studied. Second, PVL has made great progress in the treatment of breast disease, thyroid disease and even cancer. However, the understanding of its mechanism and pathway is still relatively vague, and most of them are based on clinical efficacy trials. Although there are many studies on the anti-tumor mechanism of PVL, most of them still remain in basic research, and there is no reliable evidence of clinical research. In addition, the studies on PVL almost did not involve the toxic and side effects, and the pharmacokinetics and drug interactions of PVL *in vivo* are not clear. Third, in terms of analytical techniques, it also developed from single chromatography technology to combined chromatography technology, accompanied by the emergence of high selectivity and high sensitivity detectors. Most of these methods have the advantages of fast, accurate, high sensitivity, short analysis time, strong separation ability, good selectivity, simple operation, low detection line and wide application range. This provided technical support for the component analysis and content determination of PVL. But for most methods there are still some shortcomings, such as complex sample pretreatment time-consuming, expensive equipment, reagent consumption, not conducive to health and the environment. Finally, the quality evaluation system of PVL has developed from a single active ingredient or indicator component detection system to a multi-index and multi-component detection system related to efficacy and toxicity. The fingerprints that characterize the chemical constituents of PVL are mostly HPLC or UPLC fingerprints. However, this chemical fingerprint combined with multi-component content determination method has certain limitations. For example, the concept of fingerprint is relatively vague, and only a few index components are used to quantitatively control the quality of TCM. This cannot comprehensively and reasonably evaluate the quality of TCM.

Based on the above analysis, many new problems need to be solved in the research on PVL. Further research and development are needed in the following aspects. New technologies such as identification, separation and purification of phytochemistry should be continuously used to pay attention to the biological effects and structure-activity relationships of phytochemicals. In addition to obtaining intuitive index evaluation through animal experiments and clinical experiments, new technologies and methods such as molecular biology, cell biology and histology should be combined to further explore its inherent pharmacological mechanism in order to develop low-toxic, economic and efficient innovation new drugs. The quality control of PVL should be comprehensively evaluated with chromatographic fingerprint as the core, including the acquisition of chemical composition information, the correlation study of efficacy and safety evaluation. At the same time, it is also hoped that with the continuous development of science and technology, some practical, efficient, environmentally friendly, new and cost-appropriate technologies will be widely used in the analysis of PVL components.

In summary, PVL has abundant resources and extensive pharmacological effects. In the future, with the joint efforts of scholars, PVL will continue to play an important clinical application value and great potential for drug resource development with its unique advantages, and will also have a certain development and application prospect in the beverage and health products industry.

## Glossary

PVL: *Prunella vulgaris* L
TCM: traditional Chinese medicine
HIV: human immunodeficiency virus
HSV: herpes simplex virus
CD4: cluster of differentiation 4
gp120: glycoprotein 120
MIC: minimum inhibitory concentration
BHT: butylated hydroxytoluene
SOD: superoxide dismutase
DPPH: 2,2-diphenyl-1-picrylhydrazyl
FRAP: ferric reducing antioxidant power
MDA: malondialdehyde
LPS: lipopolysaccharide
iNOS: inducible nitric oxide synthase
TNF-α: tumor necrosis factor-α
IL-1β: interleukin 1β
IL-6: interleukin 6
PGE2: prostaglandin E2
NLRP3: NACHT, LRR and PYD domains-containing protein 3
Caspase-1: cysteinyl aspartate specific proteinase 1
MFD: monocular form deprivation
AKT: attenuating the phosphorylation of protein kinase B
NF-κB: nuclear factor kappa-light-chain-enhancer of activated B
PBS: phosphate-buffered saline
MCP-1: monocyte chemoattractant protein-1
HMGB1: high-mobility group box-1
ALT: alanine aminotransferase
ALP: alkaline phosphatase
AST: aspartate transaminase
CYP2E1: cytochrome P4502E1
MHV: mouse hepatitis virus
IFN-γ: γ-interferon
TGF-β: transforming growth factor-β
BAX: Bcl-2-associated X
Bcl-2: B-cell lymphoma-2
SGLT-1: sodium glucose cotransporters 1
GLUT-2: facilitativeglucosetransporters 2
LDL: low-density lipoprotein
HDL: high-density lipoprotein
LDL-C: low-density lipoprotein cholesterol
HDL-C: high-density lipoprotein cholesterol
ICAM-1: intercellular adhesion molecules-1
VCAM-1: vascular cell adhesion molecule-1
ET-1: endothelin-1
eNOS: endothelial nitric oxide synthase
ANG II: angiotensin II
CGRP: calcitonin gene-related petide
TC: total cholesterol
TG: triglycerides
MFD: monocular form deprivation
