# Supplementary material for: Prunella vulgaris L. – A Review of its Ethnopharmacology, Phytochemistry, Quality Control and Pharmacological Effects
Source: Front Pharmacol. 2022 Jun 23;13:903171. doi: 10.3389/fphar.2022.903171 (PMC9261270; doi:10.3389/fphar.2022.903171)
Supplement: Supplementary file 1 [file DataSheet1.docx]

Table S1 Summary of the Records of *Prunella vulgaris* L.in Ancient Chinese Herbal Works

| No. | Herbal Works | Dynasty | Description | Refs |
| --- | --- | --- | --- | --- |
|  | Shen Nong’s Classic of the Materia Medica (Shén Nóng Bĕn Căo Jīng, 神农本草经) | Eastern Han Dynasty, AD 25-220 | It tastes bitter, acrid and cold. Treatment of scrofula, rat fistula, head sores, break concretions, scattered gall, qi stagnation, swollen feet, and removing dampness to relieve paralysis | (Wu) |
|  | Miscellaneous Records of Famous Physicians (Míng Yī Bié Lù, 名医别录) | Wei-Jin period, AD 220-450 | Nontoxic. Also called Yanmian. Growing in Shu County. Picking in April each year | (Tao) |
|  | Materia Medica Arranged According to Pattern (Zhèng Lèi Bĕn Căo, 证类本草) | Song Dynasty, AD 960-1279 | Treatment of eye pain caused by liver deficiency, cold tears, tendon and vein pain and eye photophobia | (Tang) |
|  | Materia Medica of South Yunnan (Diān Nán Bĕn Căo, 滇南本草) | Ming Dynasty, AD 1396-1476 | One of commonly used TCMs to treat hepatic heat, exception of liver wind, eye swelling and pain, hyperemia of conjunctiva | (Lan) |
|  | The Grand Compendium of Materia Medica (Bĕn Căo Gāng Mù, 本草纲目) | Ming Dynasty, AD 1578 | Widely distributed, mostly in wilderness, lakes and swamps, ……, Seedlings 30-60 cm tall, stems slightly square, apex spikes, 3-6 cm long, with pale purple flowers in spikes | (Li) |
|  | Enlightening Primer of Materia Medica (Bĕn Căo Méng Quán, 本草蒙筌) | Ming Dynasty, AD 1368-1644 | Occured after the winter solstice, the summer solstice exhausted | (Chen) |
|  | Essentials of Materia Medica (Bĕn Căo Bèi Yào, 本草备要) | Qing Dynasty, AD 1694 | The medicinal parts are stems and leaves, It has the effects of supplementing yang, dissipating masses and eliminating goiter | (Wang) |
|  | Materia Medica Arranged by Channel Tropism (Bĕn Căo Fēn Jīng, 本草分经) | Qing Dynasty, AD 1644-1911 | It is the first time in this book to discuss its adverse effect on human body, and to warn us that long-term use had the harm to stomach | (Yao) |
|  | Materia Medica of Combinations (Dé Pèi Bĕn Căo, 得配本草) | Qing Dynasty, AD 1644-1911 | Mild acrid, slight bitter, cold, ..., people with qi deficiency are forbidden | (Yan) |
|  | Yù Qiū Yào Jiĕ (玉楸药解) | Qing Dynasty, AD 1644-1911 | Treat scrofula, goiter, tumor, fall injury, blood collapse, white spot, sweat stain and other symptoms | (Huang) |

Table S2 Prescriptions in which *Prunella vulgaris* L. is the main component listed in Chinese Pharmacopoeia and approved by the government (Committee for the Pharmacopoeia of Chinese Pharmacopoeia Commission, 2020)

| No. | Prescription name | Type | Main Compositions | Fuction |
| --- | --- | --- | --- | --- |
| 01 | Xiasangju Keli | Granule | *Prunella vulgaris* L.;  *Chrysanthemum indicum* L.;  *Morus alba* L. and so on. | Clear liver and improve vision; Expell wind-heat and remove dampness; Relieve paralysis; Resolve sore toxin |
| 02 | YinpuJieduPian | Tablet | *Lonicera macranthoides* Hand. Mazz.;  *Taraxacum mongolicum* Hand. Mazz.;  *Chrysanthemum indicum* L.;  *Viola yedoensis* Makino;  *Prunella vulgaris* L.and so on. | Clear heat and resolve toxins |
| 03 | Qingnao Jiangya Pian | Tablet | *Scutellaria baicalensis* Georgi;  *Sophora japonica* L.;  *Prunella vulgaris* L. ;  *Achyranthes bidentata* Blume;  *Rehmannia glutinosa* Libosch. and so on. | Calm the liver and subdue yang |
| 04 | Yangxue Qingnao Keli | Granule | *Angelica sinensis* (Oliv.) Diels ;  *Paeonia lactiflora* Pall.;  *Uncariarhynchophylla* (Miq.) Miq. ex Havil.;  *Spatholobus suberectus* Dunn ;  *Prunella vulgaris* L.;  *Cassia obtusifolia* L.and so on. | Nourish blood and calm liver; Activate blood and dredge collaterals |
| 05 | Fuming Pian | Tablet | *Tribulus terrestris* L.;  *Plantago asiatica* L.;  *Equisetum hyemale* L.;  *Chrysanthemum morifolium* Ramat.;  *Prunella vulgaris* L.;  *Cassia obtusifolia* L. and so on. | Enrich and nourish liver, kidney; Nourish yin and promote body fluid; Clear the liver and improve vision |
| 06 | Huamoyan Pian | Tablet | *Prunella vulgaris* L.;  *Ligustrum lucidum* W.T.Aiton ;  *Ilexcornuta* Lindl.ex Paxt.;  *Astragalus membranaceus* (Fisch.) Bunge;  *Stephania tetrandra* S.Moore;  *Coix lacryma-jobi* var. *ma-yuen* (Rom.Caill.) Stapf  *Smilax glabra* Roxb. and so on. | Clear away heat and dampness, Promote blood circulation and dredge collaterals |
| 07 | Huamoyan Jiaonang | Granule | *Prunella vulgaris* L.;  *Ligustrum lucidum* W.T.Aiton ;  *Ilexcornuta* Lindl.ex Paxt.;  *Astragalus membranaceus* (Fisch.) Bunge;  *Stephania tetrandra* S.Moore;  *Coix lacryma-jobi* var. *ma-yuen* (Rom.Caill.) Stapf  *Smilax glabra* Roxb. and so on. | Clear away heat and dampness, Invigorate blood and dredge collaterals |
| 08 | Huamoyan Keli | Granule | *Prunella vulgaris* L.;  *Ligustrum lucidum* W.T.Aiton ;  *Ilexcornuta* Lindl.ex Paxt.;  *Astragalus membranaceus* (Fisch.) Bunge;  *Stephania tetrandra* S.Moore;  *Coix lacryma-jobi* var. *ma-yuen* (Rom.Caill.) Stapf  *Smilax glabra* Roxb. and so on. | Clear away heat and dampness, Promote blood circulation and dredge collaterals |
| 09 | Biyan Qingdu Keli | Granule | *Chrysanthemum indicum* L.;  *Paris polyphylla var. yunnanensis* (Franch.) Hand.-Mazz. ;  *Zanthoxylum nitidum* (Roxb.) DC. ;  *Xanthium sibiricum* Patrin ex Widder;  *Rubus parvifolius* L.;  *Prunella vulgaris* L.;  *Gentiana manshurica* Kitag.;  *Codonopsis pilosula* (Franch.) Nannf. and so on. | Clear heat and resolve toxins; Dissolve phlegm and dissipate masses |
| 10 | Fufang Lingjiao Jiangya Pian | Tablet | *Saiga tatarica* Linnaeus;  *Prunella vulgaris* L.;  *Scutellaria baicalensis* Georgi;  *Viscum coloratum* (Kom.) Nakai and so on. | Calm the liver and relieve heat |
| 11 | TengdanJiaonang | Capsule | *Uncaria rhynchophylla* (Miq.) Miq. ex Havil.;  *Prunella vulgaris* L.;  *Taxillus chinensis* (DC.) Danser;  *Plantago asiatica* L.;  *Sus scrofa domestica* Brisson ;  *Salvia miltiorrhiza* Bunge and so on. | Calm the liver and subdue wind; Heat Purge Fire and Nourish Yin; Relax the vessels and open the collaterals |
| 12 | Shanju Jiangya Pian | Tablet | *Crataegus pinnatifida* Bunge;  *Alisma plantago-aquatica* L.;  *Chrysanthemum morifolium* Ramat.;  *Prunella vulgaris* L.;  *Cirsium setosum* (Willd.) Besser ex M.Bieb.;  *Cassia obtusifolia* L. and so on. | Calm the liver and subdue yang |
| 13 | Xiaoying Wan | Pill | *Laminariajaponica* Aresch.;  *Sargassum pallidum* (Turn.) C.Ag.;  *Fritillaria thunbergii* Miq. ;  *Prunella vulgaris* L.;  *Meretrix meretrix* Linnaeus;  *Platycodon grandiflorum* (Jacq.) A.DC. and so on. | Eliminate stagnation and scar |
| 14 | Neixiao Luoli Pian | Tablet | *Prunella vulgaris* L.;  *Sargassum pallidum* (Turn.) C.Ag.;  *Fritillaria thunbergii* Miq.;  *Ampelopsis japonica* (Thunb.) Makino;  *Richosanthes kirilowii* Maxim.;  *Rheum palmatum* L.and so on. | Dissolve phlegm; Soften hardness; Dissipate masses |
| 15 | FufangYiganWan | Pill | *Artemisia capillaris* Thunb.;  *Isatis indigotica* Fort.;  *Gentiana manshurica* Kitag.;  *Chrysanthemum indicum* L.;  *Taraxacum mongolicum* Hand. Mazz.;  *Sophora tonkinensis* Gagnep.;  *Sedum sarmentosum* Bunge;  *Cryptotympana pustulata* Fabricius;  *Prunus armeniaca L.var.ansu* Maxim.;  Artificial bovis;  Prunella vulgaris L.and so on. | Clear heat and drain dampness; Soothe the liver and rectify the spleen; Dissolve stasis and dissipate masses |
| 16 | RupixiaoPian | Tablet | *Cervus elaphus* Linnaeus;  *Taraxacum mongolicum* Hand. -Mazz.;  *Laminariajaponica* Aresch.;  *Spatholobus suberectus* Dunn;  *Panax notoginseng* (Burk.) F. H. Chen;  *Sargassum pallidum* (Turn.) C.Ag.;  *Prunella vulgaris* L.;  *Scrophularia ningpoensis* Hemsl.and so on. | Soften hardness and dissipate masses; Invigorate blood and disperse welling-abscess; Clear heat and resolve toxins |
| 17 | RupixiaoJiaonang | Capsule | *Cervus elaphus* Linnaeus;  *Taraxacum mongolicum* Hand. -Mazz.;  *Laminariajaponica* Aresch.;  *Spatholobus suberectus* Dunn;  *Panax notoginseng* (Burk.) F. H. Chen;  *Sargassum pallidum* (Turn.) C.Ag.;  *Prunella vulgaris* L.;  *Scrophularia ningpoensis* Hemsl.and so on. | Soften hardness and dissipate masses; Invigorate blood and disperse welling-abscess; Clear heat and resolve toxins |
| 18 | RupixiaoKeli | Granule | *Cervus elaphus* Linnaeus;  *Taraxacum mongolicum* Hand. Mazz.;  *Laminariajaponica* Aresch.;  *Spatholobus suberectus* Dunn;  *Panax notoginseng* ( Burk.) F. H. Chen;  *Sargassum pallidum* (Turn.) C.Ag.;  *Prunella vulgaris* L.;  *Scrophularia ningpoensis* Hemsl.and so on. | Soften hardness and dissipate masses; Invigorate blood and disperse welling-abscess; Clear heat and resolve toxins |
| 19 | RupiSanjieJiaonang | Capsule | *Prunella vulgaris* L.;  *Ligusticum chuanxiong* Hort.;  *Bombyx mori* Linnaeus;  *Trionyx sinensis* Wiegmann;  *Bupleurum chinense* DC.;  *Paeonia lactiflora* Pall. and so on. | Promote flow of qi and blood circulation; Soften hardness and dissipate masses |
| 20 | XinnaojingPian | Tablet | *Nelumbo nucifera* Gaertn.;  *Sophora japonica* L.;  *Aucklandia lappa* Decne.;  *Prunella vulgaris* L.;  *Gentiana manshurica* Kitag.;  *Clematis chinensis* Osbeck;  *Glycyrrhiza uralensis* Fisch.and so on. | Calm the liver and subdue yang; Clear heart and tranquilization |
| 21 | Hexue Mingmu Pian | Tablet | *Typha angustifolia* L.;  *Salvia miltiorrhiza* Bge.;  *Rehmannia glutinosa* Libosch.;  *Eclipta prostrate* L.;  *Prunella vulgaris* L.;  *Scutellaria baicalensis* Georgi;  *Cassia obtusifolia* L.and so on. | Cool the blood and stanch bleeding; Enrich yin and dissolve phlegm; Nourish liver and improve vision |
| 22 | RukangWan | Pill | Ostrea gigas thunberg;  Boswellia carterii Birdw.;  Trichosanthes kirilowii Maxim.;  Prunella vulgaris L.;  Sargassum pallidum (Turn.) C.Ag.;  Astragalus membranaceus (Fisch.) Bge. and so on. | Invigorate blood and relax the liver; Dispel phlegm and soften hardness |
| 23 | RukangJiaonang | Capsule | *Ostrea gigas* thunberg;  *Boswellia carterii* Birdw.;  *Trichosanthes kirilowii* Maxim.;  *Prunella vulgaris L.*;  *Sargassum pallidum* (Turn.) C.Ag.;  *Astragalus membranaceus* (Fisch.) Bge. and so on. | Invigorate blood and relax the liver; Dispel phlegm and soften hardness |
| 24 | RukangKeli | Granule | *Ostrea gigas* thunberg;  *Boswellia carterii* Birdw.;  *Trichosanthes kirilowii* Maxim.;  *Prunella vulgaris* L.;  *Sargassum pallidum* (Turn.) C.Ag.;  *Astragalus membranaceus* (Fisch.) Bge. and so on. | Invigorate blood and relax the liver; Dispel phlegm and soften hardness |

Table S3 Chemical components isolated and identified from *Prunella vulgaris* L.

| Classification | No. | Chemical Components | Part of Herb | Ref |
| --- | --- | --- | --- | --- |
| Triterpenoids |  | Oleanolic acid | Spikes | (He et al.) |
|  |  | Ursolic acid | Spikes | (He et al.) |
|  |  | Maslinic acid | Aerial parts | (Lee et al.) |
|  |  | *β*-Amyrin | Spikes | (He et al.) |
|  |  | Hederagenin | Spikes | (Choi et al.) |
|  |  | Euscaphic acid | Spikes | (Byun et al.) |
|  |  | Tormentic acid | Whole herb | (GilSaeng) |
|  |  | Corosolic acid | Whole herb | (GilSaeng) |
|  |  | Pomolic acid | Whole herb | (GilSaeng) |
|  |  | Pyogenic acid | Spikes | (Choi et al.) |
|  |  | Betulic acid | Aerial parts | (Qi et al.) |
|  |  | Sericoside | Unknown | (Zhang and Yang) |
|  |  | Caulophyllogenin | Whole herb | (Yang and Zhao) |
|  |  | Squalene | Spikes | (Choi et al.) |
|  |  | Betulin | Unkown | (Yang et al.) |
|  |  | Arjunglcoside I | Unknown | (Zhang and Yang) |
|  |  | Vulgaside I | Spikes | (Yu et al.) |
|  |  | Vulgaside II | Spikes | (Yu et al.) |
|  |  | Niga-ichigoside F1 | Unknown | (Zhang and Yang) |
|  |  | Niga-ichigoside F2 | Unknown | (Zhang and Yang) |
|  |  | Prunelloside A | Spikes | (Zhang et al.) |
|  |  | Pruvuloside A | Unknown | (Zhang and Yang) |
|  |  | Pruvuloside B | Unknown | (Zhang and Yang) |
|  |  | Vulgarsaponin A | Spikes | (Tian et al.) |
|  |  | Vulgarsaponin B | Spikes | (Wang et al.) |
|  |  | Methyl ursolate | Leaves,Stems | (Kojima and Ogura) |
|  |  | Methyl betulinate | Roots | (Kojima et al.) |
|  |  | Methyl oleanolate | Leaves,Stems | (Kojima and Ogura) |
|  |  | Methyl maslinate | Leaves,Stems | (Kojima and Ogura) |
|  |  | 3-*Epi*-maslinic acid | Spikes | (Choi et al.) |
|  |  | Candelabrone 12-methyl ether | Spikes | (Bai et al.) |
|  |  | Methyl 3-epimaslinate | Leaves,Stems | (Kojima and Ogura) |
|  |  | Methyl 2*α*-hydroxyursolate | Leaves,Stems | (Kojima and Ogura) |
|  |  | Olean-12-ene-3*β*,28-diol | Whole herb | (Meng and He) |
|  |  | Urs-12-ene-3*β*,28-diol | Whole herb | (Meng and He) |
|  |  | Oleanolic acid 3-*O*-monoglucuronide | Spikes | (Liu et al.) |
|  |  | 3*β*-Hydroxy-urs-12-ene-28-al | Whole herb | (Meng and He) |
|  |  | 3*β*-Hydroxy-olean-12-ene-28-al | Whole herb | (Meng and He) |
|  |  | 3*β*-Hydroxyolean-5,12-diene | Spikes | (Byun et al.) |
|  |  | 3*β*,23-Dihydroxyurs-12-en-28-oic acid | Stems,Leaves | (Cai and Yan) |
|  |  | 3*β*,24-Dihydroxyursolic acid | Whole herb | (GilSaeng) |
|  |  | 3*β*,22*α*-dihydroxyolean-12-en-28-oic acid | Unkown | (Yang et al.) |
|  |  | 3*β*,22*α*-dihydroxyurs-12-en-28-oic acid | Unkown | (Yang et al.) |
|  |  | 3*β*,13*β*-dihydroxyolic-11-ene-28-oic acid | Spikes | (Du et al.) |
|  |  | 2*α*,3*α*,24-trihydroxyursa-12,20(30)-dien-28-oic acid | Spikes | (Wang et al.) |
|  |  | 2*α*,3*α*,19-trihydroxy-12-en-28-ursoic acid | Whole herb | (Gai et al.) |
|  |  | 2*α*,3*α*,23-trihydroxyursa-12,20(30)-dien-28-oic acid | Spikes | (Choi et al.) |
|  |  | 2*α*,3*α*,23- trihydroxy-12-en-28-ursolic acid | Aerial parts | (Lee et al.) |
|  |  | 2*α*,3*α*, 24-trihydroxyolean-12-en-28oic acid | Spikes | (Wang et al.) |
|  |  | 2*α*,3*α*,24-trihydroxyolean-12,20(30)-dien-28-oic acid | Fruits | (Zhou et al.) |
|  |  | 2*α*,3*α*,24-trihydroxy-12-en-28-ursolic acid | Spikes | (Wang et al.) |
|  |  | 2*α*,3*α*-24-trihydroxyursa-12-en-28-oic acid-28-*O*-*β*-*D*-glucopyranoside | Spikes | (Yu et al.) |
|  |  | 3*α*,19*α*,23,24-tetrahydroxyurs-12-en-28-oic acid | Stems,Leaves | (Cai and Yan) |
|  |  | 2*α*,3*α*,19*α*-trihydroxyurs-12-en-28-oic acid | Aerial parts | (Lee et al.) |
|  |  | 2*α*,3*α*,19*α*-trihydroxyurs-12-en-28-oic acid-28-*β*-*D*-glucopyranoside | Spikes | (Yu et al.) |
|  |  | 2*α*,3*α*,19*α*-trihydroxyurs-12-en-28-oic acid-28-*β*-*D*-glucopyranosyl-(1→2)-*β-D*-glucopyranoside | Spikes | (Yu et al.) |
|  |  | 2α,3α,19α,23-tetrahydroxyurs-12en-28-oic acid | Aerial parts | (Lee et al.) |
|  |  | 2*α*,3*α*,19*α*,24-tetrahydroxylurs-12-en-28-oic acid | Fruits | (Zhou et al.) |
|  |  | 2*α*,3*α*,19*α*,24-tetrahydroxyurs-12en-28oic acid 28-*O*-*D*-glucopyranoside | Aerial parts | (Lee et al.) |
|  |  | 2*α*,3*α*,19*α*,24-tetrahydroxy-12-en-28-ursolic acid-28-*β*-*D*-glucopyranoside | Aerial parts | (Qi et al.) |
|  |  | 2*α*,3*β*-dihydroxy-12-en-28-ursolic acid | Spikes | (Wang et al.) |
|  |  | 2*α*,3*β*-dihydroxy-12-en-28-oleanolic acid | Spikes | (Wang et al.) |
|  |  | 2*α*,3*β*,24-trihydroxy-12-en-28- oleanolic acid | Aerial parts | (Qi et al.) |
|  |  | 2*α*,3*β*,24-trihydroxy-12-en-28- ursolic acid | Whole herb | (Gai et al.) |
|  |  | 2*α*,3*β*,24-trihydroxyurs-12-en-28-oic acid-28-*β*-*D*-glucopyranoside | Spikes | (Yu et al.) |
|  |  | 2*α*,3*β*,19*α*-trihydroxyurs-12-en-28-oic acid | Spikes | (Yu et al.) |
|  |  | 2*α*,3*β*,19*α*,23-tetrahydroxy-12-en-28-oic acid-28-*β*-*D*-glucopyranoside | Aerial parts | (Qi et al.) |
|  |  | 2*α*,3*β*,19*α*,23*β*-tetrahydroxyurs-12-en-28-oic acid | Stems,Leaves | (Cai and Yan) |
|  |  | 2*α*,3*β*,19*α*,24-tetrahydroxyurs-12en-28oic acid 28-*O*-*D*-glucopyranoside | Aerial parts | (Lee et al.) |
|  |  | 2*α*,3*β*,19*α*,24-tetrahydroxyurs-12-en-28-oic acid-28-*β*-*D*-glucopyranoside | Aerial parts | (Qi et al.) |
|  |  | 5*α*,8*α*-epidioxy-(22*E*,24*R*)-ergosta-6,22-dien-3*β*-ol | Spikes | (Bai et al.) |
|  |  | 1*β*,3*β*-dihydroxyurs-12-en-28-oic acid | Stems,Leaves | (Cai and Yan) |
|  |  | Methyl 2*α*,3*α*-dihydroxyurs-12-en-28-oate | Leaves,Stems | (Kojima and Ogura) |
|  |  | Methyl 2*α*,3*α*-dihydroxyursa-12,20(30)-dien-28-oate | Roots | (Kojima et al.) |
|  |  | Methyl 2*α*,3*α*,24-trihydroxyursa-12,20(30)-dien-28-oate | Roots | (Kojima et al.) |
|  |  | Methyl 2*α*,3*α*,23-trihydroxyolean-12-en-28-oate | Leaves,Stems | (Kojima and Ogura) |
|  |  | Methyl 2*α*,3*α*,24-trihydroxyolean-12-en-28-oate | Leaves,Stems | (Kojima and Ogura) |
|  |  | Methyl 2*α*,3*α*,24-trihydroxyurs-12-en-28-oate | Roots | (Kojima et al.) |
|  |  | Methyl (12R,13S)-2*α*,3*α*,24-trihydroxy-12,13-cycle-taraxer-14-en-28-oate | Roots | (Kojima et al.) |
|  |  | Methyl (13S,14R)-2*α*,3*α*,24-trihydroxy-13,14-cycle-olean-11-en-28-oate | Roots | (Kojima et al.) |
|  |  | Methyl 2*α*,3*α*,24-trihydroxyoteana-11,13(18)-dien-28-oate | Roots | (Kojima et al.) |
|  |  | Rotundic acid 28-*O*-*α*-*D*-glueopyranosyl(1→6)-*β*-*D*-glueopyranoside | Spikes | (Yu et al.) |
|  |  | 2,3,24-trihydroxyurs-12-en-28-oic acid | Unkown | (Yang et al.) |
|  |  | 2,3,24-trihydroxyolean-12-en-28-oic acid | Unkown | (Yang et al.) |
|  |  | 3-hydroxy-11-en-11,12-dehydrogenation-28,13-oic acid lactone | Spikes | (Bai et al.) |
|  |  | 3-*O*-*α*-*L*-arabinophyranose-19*α*-hydroxyurs-12-en-28-oic acid | Stems,Leaves | (Cai and Yan) |
|  |  | 22-hydroxy-3-oxoleana-12-en-28-oic acid | Unkown | (Yang et al.) |
| Sterols |  | Stigmasterol | Spikes | (Tian et al.) |
|  |  | Spinasterone | Spikes | (Choi et al.) |
|  |  | Daucosterol | Spikes | (He et al.) |
|  |  | *α*-Spinasterol | Leaves,Stems | (Kojima and Ogura) |
|  |  | *α*-Spinasterone | Spikes | (Xu et al.) |
|  |  | *β*-Sitosterol | Whole herb | (Meng and He) |
|  |  | *β*-Daucosterol | Spikes | (Xu et al.) |
|  |  | Stigmast-7-enol | Leaves,Stems | (Kojima and Ogura) |
|  |  | Stigmast-7-en-3*β*-ol | Spikes | (Tian et al.) |
|  |  | Qingyangshengenin-3-*O*-*β*-*D*-digitoxopyranoside | Whole herb | (Lou et al.) |
|  |  | Qinyangshengenin-3-*O*-*β*-*D*-oleandropyranosyl-(1→4)-*β*-*D*-cymaropyranosyl-(1→4)-*β*-*D-*digitoxopyranoside | Whole herb | (Lou et al.) |
|  |  | 5-stigmasta-7,22-dien-3-ol | Spikes | (Choi et al.) |
|  |  | (22E, 20S, 24S)-stigmasta-7,22-diene-3-one | Whole herb | (Meng and He) |
| Phenylpropanoids |  | Caffeic acid | Spikes | (Tian et al.) |
|  |  | Chlorogenic acid | Spikes | (Liu et al.) |
|  |  | Ethyl caffeate | Spikes | (Wang et al.) |
|  |  | Caffeic acid-3-*O*-glucoside | Fruits | (Yan et al.) |
|  |  | Caffeic acid-*O*-hexoside | Unkown | (Yang et al.) |
|  |  | Scopoletin | epigeal parts | (Dmitruk) |
|  |  | Umbelliferone | epigeal parts | (Dmitruk) |
|  |  | Esculetin | epigeal parts | (Dmitruk) |
|  |  | Coniferaldehyde | Roots,Branches | (Yang and Zhao) |
|  |  | Coumarin | Whole herb | (Yang and Zhao) |
|  |  | 3,4-Dihydrocoumarin | Flowers | (Yang and Zhao) |
|  |  | Rosmarinic acid | Spikes | (Wang et al.) |
|  |  | Methyl rosmarinate | Spikes | (Wang et al.) |
|  |  | Butyl rosmarinate | Spikes | (Wang et al.) |
|  |  | Ethyl rosmarinate | Spikes | (Wang et al.) |
|  |  | Salviaflaside | Unkown | (Yang et al.) |
|  |  | Sinapaldehyde | Roots,Branches | (Yang and Zhao) |
|  |  | *trans*-Salviaflaside methyl ester | Fruits | (Yan et al.) |
|  |  | *trans*-Salviaflaside | Fruits | (Yan et al.) |
|  |  | Danshensu | Spikes | (Gu et al.) |
|  |  | Methyl 3,4-dihydroxyphenyl lactate | Whole herb | (Gai et al.) |
|  |  | Ethyl 3,4-dihydroxyphenyl lactate | Whole herb | (Gai et al.) |
|  |  | 4-Allyl-2,6-dimethoxyphenol | Roots,Branches | (Yang and Zhao) |
|  |  | *p*-Hydroxycinnamic acid | Aerial parts | (Lee et al.) |
|  |  | *p*-Coumaric acid | Spikes | (Wang et al.) |
|  |  | 3,4,*α*-trihydroxy-butyl phenylpropionate | Spikes | (Wang et al.) |
|  |  | 3,4,*α*-trihydroxy-methyl phenylpropionate | Spikes | (Wang et al.) |
|  |  | (-)-syringaresinol-4-*O*-*β*-*D*-glucopyranoside | Fruits | (Yan et al.) |
|  |  | 2-hydroxy-3-(3',4'-dihydroxyphenly)propanoic acid | Aerial parts | (Lee et al.) |
| Flavonoids |  | Rutin | Flowers | (Wang et al.) |
|  |  | Kaempferide | Spikes | (Liu et al.) |
|  |  | Quercetin | Spikes | (Wang et al.) |
|  |  | Quercitrin | Spikes | (Yu et al.) |
|  |  | Luteolin | Epigeal parts | (Dmitruk et al.) |
|  |  | Cynaroside | Epigeal parts | (Dmitruk et al.) |
|  |  | Wogonin | Spikes | (Xu et al.) |
|  |  | Homoorientin | epigeal parts | (Dmitruk et al.) |
|  |  | Hesperidin | Flowers | (Wang et al.) |
|  |  | Syringaldehyde | Roots,Branches | (Yang and Zhao) |
|  |  | Quercetin-3-*O*-*β*-*D*-galactoside | Spikes | (Wang et al.) |
|  |  | Quercetin-3-*O-β*-*D*-glucoside | Flowers | (Wang et al.) |
|  |  | Quercetin-3-*O*-*β*-*D*-glucopyranoside | Aerial parts | (Lee et al.) |
|  |  | Quercetin-3-*O*-glucoside | Unkown | (Zhang and Yang) |
|  |  | Quercertin 3-*O*-*α*-L-rhamnopyranosyl (1→6)-*β*-*D*-glucopranoside | Aerial parts | (Lee et al.) |
|  |  | Kaempferol-3-*O*-glucoside | Unkown | (Zhang and Yang) |
|  |  | Kaempferol-3-*O*-*β*-*D*-glucoside | Whole herb | (Gai et al.) |
|  |  | Kaempferol 3-*O*-*β*-*D*-glucopyranoside | Aerial parts | (Lee et al.) |
|  |  | Kaempferol 3-*O*-*α*-*L*-rhamnopyranosyl(1→6)-*β*-*D-*glucopranoside | Aerial parts | (Lee et al.) |
|  |  | Acacetin-7-*O*-*β*-*D*-glucopyranoside | Spikes | (Zhang et al.) |
| Organic acids |  | Citric acid | Spikes | (Liang et al.) |
|  |  | Ferulic Acid | Spikes | (Liu et al.) |
|  |  | Gluconic acid | Spikes | (Liang et al.) |
|  |  | Protocatechuic acid | Spikes | (Liang et al.) |
|  |  | Benzoic acid | Flowers, Branches | (Yang and Zhao) |
|  |  | Gallic acid | Unkown | (Yang et al.) |
|  |  | Malic acid | Spikes | (Liang et al.) |
|  |  | Malonic acid | Whole herb | (Yang and Zhao) |
|  |  | Cyclopentaneacetic acid | Spikes | (Bai et al.) |
|  |  | Octanoic acid | Roots, Flowers,Leaves | (Yang and Zhao) |
|  |  | Pentadecanoic acid | Roots,Branches | (Yang and Zhao) |
|  |  | Palmitic acid | Spikes | (Tian et al.) |
|  |  | Palmitic acid ethyl ester | Spikes | (Tian et al.) |
|  |  | Stearic acid | Spikes | (Tian et al.) |
|  |  | Oleic acid | Spikes | (Tian et al.) |
|  |  | Arachidic acid | Spikes | (Tian et al.) |
|  |  | Behenic acid | Spikes | (Tian et al.) |
|  |  | Palmitoleic acid | Roots | (Yang and Zhao) |
|  |  | Monopalmitin | Flowers,Leaves | (Yang and Zhao) |
|  |  | 4-Hydroxybenzoic acid | Unkown | (Zheng) |
|  |  | Linolenic acid | Flowers,Leaves | (Yang and Zhao) |
|  |  | Linoleic acid | Branches | (Yang and Zhao) |
|  |  | Dehydroabietic acid | Roots | (Yang and Zhao) |
|  |  | Methyl linolenate | Roots, Branches | (Yang and Zhao) |
|  |  | Ellagic acid-*O*-hexoside | Unkown | (Yang et al.) |
|  |  | Vinyl crotonate | Roots | (Yang and Zhao) |
|  |  | Dimethyl fumarate | Flowers,Leaves,Branches | (Yang and Zhao) |
|  |  | Methyl-ellagic acid-*O*-pentoside | Unkown | (Yang et al.) |
|  |  | Methyl 3,4-dihydroxybenzoate | Unkown | (Zheng) |
|  |  | Gentisic acid 5-*O*-*β*-*D*-(6'-salicylyl)-glucopyranoside | Spikes | (Gu et al.) |
| Volatile oils |  | Myrcene | unknown | (Yang et al.) |
|  |  | Cuparene | Aerial parts | (Morteza-Semnani et al.) |
|  |  | (-)-Thujopsen | Roots | (Yang and Zhao) |
|  |  | Germacrene D | Aerial parts | (Morteza-Semnani et al.) |
|  |  | Spathulenol | Aerial parts | (Morteza-Semnani et al.) |
|  |  | *α*-Patchoulene | Aerial parts | (Morteza-Semnani et al.) |
|  |  | *α*-Cedrene | Aerial parts | (Morteza-Semnani et al.) |
|  |  | *α*-Chamigrene | Aerial parts | (Morteza-Semnani et al.) |
|  |  | *α*-Calacorene | Roots | (Yang and Zhao) |
|  |  | *α*-Phellandrene | unknown | (Yang et al.) |
|  |  | *α*-Muurolol | Aerial parts | (Morteza-Semnani et al.) |
|  |  | *α*-Pinene | Aerial parts | (Morteza-Semnani et al.) |
|  |  | *β*-Pinene | unknown | (Yang et al.) |
|  |  | *β*-Bisabolol | Aerial parts | (Morteza-Semnani et al.) |
|  |  | *β*-Curcumene | Roots | (Yang and Zhao) |
|  |  | *β*-Copaene | Aerial parts | (Morteza-Semnani et al.) |
|  |  | *β*-Bourbonene | Aerial parts | (Morteza-Semnani et al.) |
|  |  | *β*-Elemene | Flowers | (Yang and Zhao) |
|  |  | *δ*-Amorphene | Aerial parts | (Morteza-Semnani et al.) |
|  |  | *D*-Limonene | Spikes | (Yang) |
|  |  | Vanillin | Roots,Flowers,Branches | (Yang and Zhao) |
|  |  | Vanillin methyl ketone | Branches | (Yang and Zhao) |
|  |  | Sinapaldehyde | Roots,Branches | (Yang and Zhao) |
|  |  | Dodecyl aldehyde | Spikes | (Wang et al.) |
|  |  | Ferruginol | Roots | (Yang and Zhao) |
|  |  | *cis*-Isoeugenol | Roots,Branches | (Yang and Zhao) |
|  |  | 4-Vinylguaiacol | Whole herb | (Yang and Zhao) |
|  |  | Phytol | Roots,Flowers,Leaves | (Yang and Zhao) |
|  |  | Isophytol | Leaves | (Yang and Zhao) |
|  |  | Phytone | Flowers,Leaves,Branches | (Yang and Zhao) |
|  |  | Geranylgeraniol | Roots | (Yang and Zhao) |
|  |  | Geranyl linalool | Roots | (Yang and Zhao) |
|  |  | Linalool | Unkown | (Yang et al.) |
|  |  | Linalyl acetate | Unkown | (Yang et al.) |
|  |  | Citronellyl propionate | Roots,Branches | (Yang and Zhao) |
|  |  | Methyl salicylate | Flowers,Leaves,Branches | (Yang and Zhao) |
|  |  | Bornyl acetate | Aerial parts | (Morteza-Semnani et al.) |
|  |  | *E*-Ocimenone | Aerial parts | (Morteza-Semnani et al.) |
|  |  | (*Z*)-*β*-Damascenone | Aerial parts | (Morteza-Semnani et al.) |
|  |  | *(E)*-*β*-Ionone | Aerial parts | (Morteza-Semnani et al.) |
|  |  | *cis*-Eudesma-6,11-diene | Aerial parts | (Morteza-Semnani et al.) |
|  |  | *cis*-Sesquisabinene hydrate | Aerial parts | (Morteza-Semnani et al.) |
|  |  | 1,10-di-*epi*-cubenol | Aerial parts | (Morteza-Semnani et al.) |
|  |  | 1,8-Cineol | Unkown | (Yang et al.) |
|  |  | Isochiapin B | Spikes | (Wang et al.) |
|  |  | *n*-Pentadecane | Spikes | (Yang) |
|  |  | *n*-Eicosane | Aerial parts | (Morteza-Semnani et al.) |
|  |  | *n*-Heneicosane | Spikes | (Wang et al.) |
|  |  | *n*-Pentacosane | Aerial parts | (Morteza-Semnani et al.) |
|  |  | *n*-Hexacosane | Aerial parts | (Morteza-Semnani et al.) |
|  |  | *n*-Heptacosane | Aerial parts | (Morteza-Semnani et al.) |
|  |  | *n*-Octacosane | Aerial parts | (Morteza-Semnani et al.) |
|  |  | *n*-Nonacosane | Aerial parts | (Morteza-Semnani et al.) |
|  |  | *n*-Tetratriacontane | Spikes | (Yang) |
|  |  | *n*-Hexatriacontane | Spikes | (Wang et al.) |
|  |  | *n*-Tetracontane | Spikes | (Wang et al.) |
|  |  | Sesquicineol-2-one | Aerial parts | (Morteza-Semnani et al.) |
|  |  | Selin-11-en-4-*α-*ol | Aerial parts | (Morteza-Semnani et al.) |
|  |  | Phenylacetaldehyde | Whole herb | (Yang and Zhao) |
|  |  | *m*-Dimethylbenzene | Spikes | (Yang) |
|  |  | 1,2-Benzenedicarboxylic acid | Spikes | (Yang) |
|  |  | Hexanal | Aerial parts | (Morteza-Semnani et al.) |
|  |  | Decyl aldehyde | Roots | (Yang and Zhao) |
|  |  | Neophytadiacid | Spikes | (Wang et al.) |
|  |  | Isochiapin B | Spikes | (Wang et al.) |
|  |  | 1,2 Dibutyl phthalate | Spikes | (Wang et al.) |
|  |  | 9-Octadecene | Spikes | (Wang et al.) |
|  |  | 1,6-Cyclodeconediene | Spikes | (Wang et al.) |
|  |  | 1,1-Dimethoxy-hexadecane | Spikes | (Wang et al.) |
|  |  | 1,1'-Dioxy-dodecane | Spikes | (Wang et al.) |
|  |  | 6,10-xylene-2-undecanone | Spikes | (Wang et al.) |
| Saccharides |  | Glucose | Inflorescence | (Tabba et al.) |
|  |  | Galactose | Inflorescence | (Tabba et al.) |
|  |  | Xylose | Inflorescence | (Tabba et al.) |
|  |  | Arabinose | Whole herb | (Zhang) |
|  |  | Rhamnose | Whole herb | (Zhang) |
|  |  | Mannose | Whole herb | (Zhang) |
| Others |  | Tanshinone I | Spikes | (Gu et al.) |
|  |  | Aurantiamide acetate | Spikes | (Gu et al.) |
|  |  | Rhein | Spikes | (Gu et al.) |
|  |  | Chrysophanic acid | Spikes | (Xu et al.) |
|  |  | 2-Hydroxy-3-methylanthraquine | Spikes | (Xu et al.) |
|  |  | Cytidine | Spikes | (Zhou et al.) |
|  |  | Protocatechualdehyde | Spikes | (Yang et al.) |

Table S4 Pharmacology effects of  *Prunella vulgaris* L.

| Pharmacology effects | Extract/Compound/Formule | Type of disease | Model/Cell type | Observation | Administration | Dosage | Outcome | Description | Refs |
| --- | --- | --- | --- | --- | --- | --- | --- | --- | --- |
| Anti-tumor | Extract | Lymphoma | Jurkat human T lymphoma cells | *In vitro* |  | 15, 20 or 25 μg/mL | Remarkably inhibited the proliferation of Jurkat cells with an IC50 of 20.23 ± 0.31 μg/mL ; and apoptosis ratio increased gradually compared to control group (P < 0.05) | Induced apoptosis of Jurkat cells by down-regulating Bcl-2 protein and up-regulating Bax protein | (Chen et al.) |
|  | Polysaccharides | Lung adenocarcinoma | Male C57BL/6 mice subcutaneously inoculated with subcutaneous injection of 10^6^ Lewis cells in 200 μL PBS in the right anterior limb | *In vivo* | Administered intragastrically once a day for 14 consecutive days | 37.5 or 75 mg/mL | High-dose group significantly enhanced the thymus index (p≤0.01, 46.25 ± 7.44 vs 35.71 ± 7.87) and spleen index (p≤0.01, 192.50 ± 26.05 vs 155.71 ± 16.18) in tumor-bearing mice as compared to saline group | Enhanced the immune response in tumor-bearing mice | (Feng et al.) |
|  | 60% Ethanol extract | Non-small cell lung cancer (NSCLC) | Female A/J mice received benzo[a]pyrene in corn oil (100 mg/kg) by intraperitoneal injection; SPC-A-1 cells | *In vivo*, and i*n vitro* | Administered orally everyday for 24 weeks | 10 g/kg; 125, 250 or 500 μg crude drug/mL | The number of tumors was lower than untreated group (31.2 ±5.66 vs 3.0 ± 2.16, p≤0.01); Treatment with P-60 decreased the tumor multiplicity by 90.3%; the proportion of apoptotic cells increased significantly (5.7 % in blank group vs 37.0 % 500μg in crude drug / mL group) | Promoting apoptosis and regulating the cell cycle | (Feng et al.) |
|  | Oleanolic Acid | Lung adenocarcinoma | Human lung adenocarcinoma cell line SPC-A-1 | *In vitro* |  | 4, 8 or 16 μM | Enhanced proliferation inhibiting rate ( 20.3 ± 2.99% in 1μM group vs 82.1 ± 1.57% in 64 μM group); Significantly promoted cell apoptosis compared with normal and 1% DMSO group (p ≤ 0.01) | Upregulating the expressions of Bax and Bad proteins while downregulating the expression of Bcl-2 protein | (Feng et al.) |
|  | Aqueous extract | Hepatocellular carcinoma | Human liver carcinoma HepG2, Huh-7 and Hep3B cells | *In vitro* |  | 5, 10 mg/ mL | Affected migration and invasion of human liver carcinoma cells | Inhibiting activities of metalloproteases, MMP-2 and MMP-9, without affecting cell viabilities | (Kim et al.) |
|  | Acetate extract (endophytic fungus xkc-s03) | Gastric cancer | Male nude BALB/c mice subcutaneously injected with SGC-7901 cells (5x10^6^ in 0.5 mL 0.9% saline) in the flanks ; SGC-7901 cell line | *In vivo*, and *in vitro* | Administered intraperitoneally daily for 15 days | 50, 100 mg/kg；25, 50, 100, 250 or 500 µg/ mL | Inhibited cell proliferation ;Treated with S03-EA group demonstrated inhibition of tumor volume growth by 17.91 and 65.67%, and of tumor weight growth by 25.70 and 64.79%, respectively | Upregulating Bax expression, downregulating Bcl-2 expression and the suppression of VEGF expression | (Tan et al.) |
|  | Hyperoside | Non-small cell lung cancer | Human A549 cells | *In vitro* |  | 10, 50 or 100 µM | Inhibited the cells proliferation and induced apoptosis of the cells | Elevated the protein phosphorylation levels of p38 MAPK and JNK, disrupted MMP, facilitated the release of cytochrome c and AIF into cytoplasm, activated capase-9 and caspase-3 | (Yang et al.) |
|  | Ethanol extract | Colorectal cancer | Human colon carcinoma HCT-8 cells | *In vitro* |  | 0.25, 0.5 or 1.00 mg/mL | Significantly decreased cell proliferation and viability, in a dose-dependent manner, induced apoptosis of cells | The expression level of miR-34a was upregulated, whereas expression levels of its target genes Notch1, Notch2 and Bcl-2 were downregulated | (Fang et al.) |
|  | Supercritical fluid extracts (CO_2_) | Uterine myoma | Human uterine smooth muscle cells (HUSMCs) and human uterine myoma cells (HUMCs) | *In vivo*, and *in vitro* |  | 0.65, 1.30, 1.95, or 2.60 mg/ mL; 0.11, 0.22 or 0.44 g/kg | Promoted the apoptosis of the cells, and inhibited the transition of UM cells from the G0/G1 stage into the G2 stage, decreased the concentrations of estrogen and progesterone | Downregulating the expression levels of the survivin and Bcl-2 proteins and upregulating the expression levels of caspase-3 and Bax through the mitochondria-mediated apoptotic pathway. | (Lin et al.) |
|  | Total flavonoids | Hepatocarcinoma | Kunming male mice inoculated subcutaneouslys with 0.2 ml ascites tumor solution of H22 cells in the armpit of the left forelimb, Human hepatocarcinoma SMMC-7721 cells | *In vivo*, and *in vitro* | Indicated weight adjusted dose by gavage once a day for 14 days | 50, 100 or 200 mg/kg; 100, 200, 400, 800 μg/mL | Had an obvious anti-hepatocarcinoma effect, Inhibited the activity of SMMC-7721 cells, and reduced the tumor volume and weight in H22 tumor bearing mice. inhibited liver metastasis of H22 tumor | Through promoting apoptosis related to activation of the PI3K/Akt/mTOR pathway and inhibiting autophagy of liver tumor cells | (Song et al.) |
|  | Aqueous extract | Papillary thyroid carcinoma | Female BALB/c nu/nu mice subcutaneously injected K1 cells into the right flank; The human PTC cell line and normal human thyroid follicular epithelial cells HUM-CELL-0097 | *In vivo*, and *in vitro* | Administered orally by gavage once a day for 14 days | 0.025, 0.05 or 0.1 g/kg; 0.05, 0.1, 0.2, 0.4, 0.8, or 1.6 mg/mL | Suppressed tumor growth accompanied by improvements in autophagy-related protein expressions in xenografts, inhibited cell growth and induced autophagic flux | Autophagy associated with AMPK/mTOR/ ULK1 pathway inhibited PTC growth in vitro and in vivo | (Song et al.) |
|  | Methanolic extract | Breast carcinoma | Female immunodeficient nude mice injected with 5×10^6^ MCF-5 cells subcutaneously at flanks, MCF-5 cell line | *In vivo,* and *in vitro* | Injected intraperitoneally three times a week | 25 mg/kg; 12.5, 25 or 50 μg/mL | Inhibited tumor weight and volume of xenografted tumors, triggered apoptosis cell death associated with high expression of Bax and low expression of Bcl-2, caused arrest of the cells in the G2/M phase | Via apoptosis induction, inhibition of angiogenesis, cell cycle arrest, and modulation of PI3K/AKT signaling pathway. | (Gao et al.) |
|  | Aqueous extract | Cholangiocarcinoma | Human QBC939, RBE cholangiocarcinoma cells | *In vitro* |  | 50, 100 or 200 μg/mL | Proliferation inhibition rate of cancer cells treated with 100 and 200 μg/ml increased in a time and concentration dependent manner. Treated with 100μg/ml, cancer cells showed typical apoptotic morphological changes | Markedly inhibited the proliferation and induced apoptosis of cancer cells | (Wu and Xu) |
|  | Combined with cyclophosphamide | Osteosarcoma | Kunming mice, half male and half female, subcutaneously inoculated with 0.1 mL S180 peritoneal subculture cells in the right axillary | *In vivo* | Administered orally by gavage once a day for 14 days | 1 or 2 g/kg | The tumor inhibition rate and apoptosis rate in the combined chemotherapy group were significantly higher ( P < 0.05 ), and the histopathological changes and the changes of related proteins and genes were more obvious, compared with those in the model and other administration groups | Down-regulating the expression of RANK and RANKL in OPG/RANK/ RANKL signaling pathway and up-regulating the expression of OPG and OPG/RANKL | (Du et al.) |
|  | Granules | Glioma | Male BALB/cA-nu mice subcutaneously injected with U87 cells ( 1 × 106 cells / 100 μL ) in the lateral abdomen; Glioma U87 cells | *In vivo,* and *in vitro* | Administered orally by gavage once every other day for 30 days | 1.8, 3.6 or 5.4 g/kg; 0.5, 1 or 2 mg/mL | Compared with the control group, the number of tumor cell colonies decreased and the number of apoptotic cells increased in the PVL treatment group (P < 0.05), with a dose-dependent increase trend | Through regulating the expression of Bax mRNA and protein, Bcl-2 mRNA and protein, Caspase-3 mRNA, Caspase-9 mRNA, Cleaved-Caspase-3, and Cleaved-Caspase-9 | (Du et al.) |
|  | Granules | Cervical cancer | Cervical cancer HeLa cells | *In vitro* |  | 40 μg/mL | The inhibition rate of cell proliferation was significantly increased, the number of invasion was significantly reduced, and the expression level of E-cadherin protein was significantly increased, compared with the control group ( P < 0.01 ). | Inhibited proliferation, invasion and EMT of cervical cancer cells by up-regulating miR-34b expression | (Wang et al.) |
|  | Extract | Esophageal cancer | Esophageal cancer Eca-109 cell line | *In vitro* |  | 168 μg/mL | Proliferation rate was ( 43.53 ± 0.42 % vs 38.21 ± 0.34 % ) and apoptosis rate was ( 38.52 ± 0.42 % vs 35.41 ± 0.36 % ), compared with positive control group ( P < 0.01 ) | Reduceed the level of Survivin expression，increaseed the level of Caspase-3 expression and induced apopltosis | (Zheng et al.) |
|  | Rosmarinic acid | Colorectal carcinoma | Human Colon Cancer Cell Line Ls174-T cells; Male C57BL/6 mice subcutaneously injected with Lewis lung carcinoma (LLC) cells (10^5^ cells in 200 μL of PBS ) in the flanks | *In vivo,* and *in vitro* | Administered by celiac injection for 20 days | 1, 2 and 4 mg/kg; 20, 40, 60 and 80 μg/mL | Intraperitoneal administration of 2mg of rosmarinic acid reduced weight of tumors and the number of lung nodules significantly compared with those of control group | Showed anti-invasion effect via the Extracellular Signal-Regulated Kinase and Oxidation–Reduction Pathway in Ls174-T Cells | (Xu et al.) |
| Anti-viral | Extract | Human immunodeficiency virus | Lymphoid cell line MT-4; Monocytoid cell line u937; Humanperipheral blood mononuclear cells | *In vitro* |  | 6, 30, and 12.5μg/mL | Inhibited HlV-1 replication Blocked HIV-1 cell-to-cell transmission, prevented syncytium formation and intertered with the ability of both HIV-1 and purified gp120 to bind to CD4 | Antagonizes HV-1 infection of susceptible cells by preventing infection attachment to the CD4 receptor | (Yao et al.) |
|  | Polysaccharide( PPS-2b) | Herpes simplex virus | Vero cells; HSV-1 skin lesion model in guinea pigs and HSV-2 genital infection model in BALB/c mice | *In vivo,* and *in vitro* | Skin and vaginal administration respectively | 1.5 g or 2 mg per animal; 10, 20, 30, 40 and 50 μg/mL | Guinea pigs treated with the PVL oniment showed a significant reduction (P< 0.01) in skin lesions, and Mice received PVL cream treatment showed a significant reduction (P< 0.01) in mortality | Inactivated HSV-1 directly, blocked HSV-1 binding to Vero cells, and inhibited HSV-1 penetration into Vero cells | (Zhang et al.) |
|  | Aqueous extract | SARS-coronavirus 2 | The human embryonic kidney cells (HEK293T) and kidney epithelial cells (VeroE6 and Vero cells) | *In vitro* |  | 25, 50, and 100 μg | Displayed potent inhibitory effects on SCoV-2 SP (including SPG614 mutant) pseudotyped virus (SCoV-2-SP-PVs) mediated infections | Directly interrupt SCoV2–SP binding to its receptor ACE2 and block the viral entry step | (Ao et al.) |
|  | Aqueous extract | Ebola virus | Human Umbilical Vein Endothelial cells (HUVECs), kidney epithelial cells extracted from African green monkey (VeroE6)， Human PBMC derived macrophages | *In vitro* |  | 0.78, 1.56, 3.13, 6.25, 12.5, 25, 50, and 100 μg/mL | Displayed inhibitory effect on EBOV-GP pseudotyped virus (EBOV-GP-V)-mediated infectionin HUVEC and macrophage cell lines, blocked an eGFP-expressing Zaire ebolavirus (eGFP-ZEBOV) infection inVeroE6 cells | Binding directly to EBOV-GP-Vs and blocking the early viral event | (Zhang et al.) |
|  | Aqueous extract | Zika Virus | IFNAR1-/C57BL/6 mouse model of Zika virus infection; HeLa cell lines, kidney epithelial cells extracted from African green monkey (Vero) | *In vivo,* and *in vitro* | Continuous intragastric administration for 12 d, once a day | 450 and 900 mg/kg; 0.5, 1, 1.5, 2, and 2.5 μg/mL | Improved the body quality of mice, increased the survival rate, reduced the number of serum viral RNA copies, and the death protection rate of mice in the high dose group (900 mg/kg) could reach 80 %. the EC_50_ of the aqueous extract of PVL against Zika virus was (51.32–2.13) μg/mL, SI = 48.71) | Inhibited the cell lesions caused by Zika virus, inhibited the nucleic acid replication and protein expression of Zika virus, Directly killing Zika virus or inhibiting virus recognition and entry into host cells | (Li et al.) |
| Anti-inflammatory | Aqueous extract | Myopia | Male Golden Syrian hamsters model of myopia; Human RPE (H-RPE) cell, ARPE-19 cell | *In vivo,* and *in vitro* | Eye drops (10 μL) were applied topically to both eyes of the hamsters twice a day | 150 ng/mL; 10, 20, 30, 40, 60, 80, 100 and 120 μg/mL | Reduced IL-6, IL-8, and TNF-α expression in RPE cells. inhibited myopia-related TGF-β1, MMP-2, NF-κB expression while increasing type Ⅰ collagen expression in MFD induced hamster model | Inhibited inflammation by attenuating the phosphorylation of AKT and NF-κB pathway | (Lin et al.) |
|  | Aqueous extracts | Experimental Autoimmune Thyroiditis | Female Lewis rats; Nthy-ori 3–1 cell | *In vivo,* and *in vitro* | Administered orally by gavage once a day for 12 weeks | 5 μmol/L; 2 mL/kg | The thyroid volume, thyroiditis inflammation score and serum thyroglobulin antibody levels of EAT rats were attenuated (P<0.01); significantly reduced the elevated levels of the proinflammatory cytokines TNF-α, IL-6, IL-1β and MCP-1 both *in vivo* (P<0.01) and *in vitro* (P<0.05). | Decreased the activity of the TLR9/MyD88 pathway and proinflammatory cytokines through HMGB1 | (Guo et al.) |
| Hypotensive, Hypoglycemic and Hypolipidemic | Ethanol extract | Diabetes | Streptozotocin-induced diabetic ICR mice | *In vivo* | Regular gavage once a day for 28 days | 8, 4g /kg | Reduced weight loss and excessive drinking in diabetic ICR mice. Significantly reduced the serum triglyceride, cholesterol, LDL content in diabetic mice, increase HDL content | Showed that PVE had good hypoglycemic potential | (Li et al.) |
|  | Aqueous extracts | Type 2 diabetes | Caco-2 cell | *In vitro* |  | 0.25, 0.5 and 0.75 μg/mL | Delayed carbohydrate hydrolysis and influence glucose uptake, lowering postprandial blood glucose levels | Decreased the expression of α-glycosidase, SGLT-1, GLUT-2 and Na^+^-K-ATP enzyme in Caco-2 cells | (Wu et al.) |
|  | Aqueous extracts | Diabetic Atherosclerosis | Male db/db mice, Male W.T. mice | *In vivo* | Orally administered once a day in the drinking water | 100 mg/kg and 200 mg/kg | Markedly lowered blood glucose and systolic blood pressure, total plasma cholesterol, triglyceride, and LDL-cholesterol; also increased the HDL-cholesterol | Improvement of the developed diabetic symptoms and the vascular dysfunction | (Hwang et al.) |
|  | Active fraction from compound PVL ( AFCP) | Hypertension | Isolated Thoracic Aorta of SD Ｒats | *In vitro* |  | 100, 150, 200, 250 and 300 μg/mL | Induced significant relaxation in aorta rings pre-contracted by phenylephrine，and the relaxation effect was significant (75% ±8%) in endothelium-intact aortic and endotheliumdenuded aortic | Might be related to the inhibition of intracellular calcium release and extracellular calcium influx | (Que et al.) |
|  | Combined with *Uncaria rhynchophylla* | Primary hypertension | Spontaneous hypertension WKY rats | *In vivo* | Intragastric administration once daily for 4 weeks | 2.1 g/kg | The serum ANG II, ET content and left ventricular mass index of rats in the PVL combined with *Uncaria rhynchophylla* group were lower than those in the model group (P< 0.05), and the CCRP content was higher than that in the model group (P<0.05) | The decrease of ANG II, and ET content in serum and the increase of CGRP content in serum | (Wang et al.) |
| Hepatoprotective | Total triterpenoids | CCl_4_-induced acute liver injury | Rat model of acute liver injury | *In vivo* | Intragastric administration for 6 days | 62.5, 125 and 250 mg/kg | Reduced the activities of ALT and AST in serum of rats with acute liver injury, reduce the level of MDA in liver homogenate, increase the levels of SOD and GSH-Px, and inhibit the expression of CYP2E1 in liver tissue; the pathological changes in liver tissue of rats were significantly reduced | Anti-lipid peroxidation and inhibition of CYP2E1 expression | (Zhang et al.) |
|  | 80 % methanol extract | Alcohol-induced liver injury | Alcohol-induced oxidative stress Wistar rat model of liver injury | *In vivo* | Intragastric administration once a day for 28 days | 1.575 g/kg | Significantly improved the metabolic disorder of liver injury induced by alcohol, and significantly reduced the contents of inflammatory factors (TNF-α, IL-6, IL-1β) and liver function marker enzymes (ALT, AST, ALP) in serum | Regulated by phenylalanine, tyrosine, and tryptophan biosynthesis pathways | (Deng et al.) |
|  | Total triterpenoids | Fulminant hepatic failure | Model of fulminant hepatic failure in mice | *In vivo* | Gavage intervention | 100 μg/mL | The levels of serum inflammatory factors IL-2, IL-6 and IL-10 and the abnormal expressions of ERK pathway proteins Ras, Raf, MEK and ERK1/2 were significantly decreased (P< 0.05) compared with those in the model group | Inhibited over-activated MEK/ERK signaling pathway and inflammatory response | (Cui and Ren) |
|  | Capsule | Autoimmune hepatitis | Autoimmune hepatitis model of C57BL/6 mice | *In vivo* | Daily gavage for 1 month | 50, 100, 200 mg /kg | The liver necrosis and inflammatory cell infiltration were decreased, the expressions of pro-inflammatory cytokines IFN-γ and IL-17A were decreased, the expression of anti-inflammatory cytokines TGF-β was increased, and the expressions of BAX and caspase-3 were decreased dose-dependently | Improved AIH symptoms in mice through anti-inflammation and anti-apoptosis | (Tian et al.) |
| Others | Total flavonoids | Osteoarthritis | Osteoarthritis SD rat model | *In vivo* | By gavage once a day for 8 weeks | 6.125 12.5, 24.5 mg /kg | Compared with the OA group, the tenderness threshold and heat pain threshold of rats were significantly increased，and the Mankin 's score， serumTNF-α, IL-13 levels, chondrocyte apoptosis number, cartilage tissue Caspase-3, RhoA and ROCK protein expression were significantly reduced | Improved rat articular cartilage damage by inhibiting chondrocyte apoptosis inosteoarthritis rats，which may be related to the inhibition of RhoA/ROCK signaling pathway | (Lu et al.) |
|  | Total flavonoids | Osteoporosis | Ovariectomized, osteoporotic SD rat model | *In vivo* | By gavage once a day for 12 weeks | 10%, m/m | The level of ALP, number of osteoclasts and bone resorption perimeter percentage were lower in the flavonoids group than in the control group (p<0.05); the levels of OPG, BMD, and the relative volume and thickness of trabecular bone were higher (p<0.05) in the flavonoids group than in the control group | Enhanced osteoblast function, decreased bone resorption and bone metabolism, increased bone formation, reduced trabecular bone loss, and inhibited the reduction in bone mass and bone strength | (Liu et al.) |

Table S5 Analytical methods of *Prunella vulgaris* L.

| NO. | Analytical methods | Analysis components | Description | Ref |
| --- | --- | --- | --- | --- |
|  | Headspace solid-phase microextraction (HS-SPME) combined with gas chromatography-mass spectrometry (GC-MS) | Volatile components | This method has good selectivity and high efficiency. It also makes up for the shortcomings of traditional extraction methods, such as steam distillation, soxhlet extraction and solvent extraction, which are time-consuming, inefficient, toxic solvent residual and even lead to the degradation of sensitive compounds and potential hydrolysis reactions. | (Yang et al.) |
|  | High performance liquid chromatography coupled with mass spectrometry (HPLC-UV coupled with MS) | Triterpenoids, aromatic compounds, phenolic compounds and anthraquinones | The results show that this method is effective and reliable. It makes up for the shortcomings of low resolution and low sensitivity of capillary zone electrophoresis (CZE), meets the requirements of simultaneous determination of multiple components, and provides a certain basis for PV quality evaluation | (Liu et al.) |
|  | High performance liquid chromatography coupled with time-of-flight mass spectrometry (HPLC-QTOF-MS/MS); High performance liquid chromatography combined with evaporative light scattering detection (HPLC-ELSD) | Rosmarinic acid, maslinic acid, corosolic acid, betulin, oleanolic acid, and ursolic acid | This method can simultaneously carry out qualitative and quantitative analysis, and also has the advantages of high sensitivity, high selectivity, high precision and high information acquisition speed. However, there are also some shortcomings. For example, the analysis of unknown structural compounds is still insufficient, the ion source replacement is difficult, and there are many problems in the supporting liquid system | (Yang et al.) |
|  | High-performance liquid chromatography tandem triple quadrupole mass spectrometry (HPLC-QTRAP-MS/MS) | Nine phenolic acids, three coumarins, eight flavonoids and one pentacyclic triterpenoid | This method has obvious advantages in analysis speed, resolution and sensitivity, and the solvent consumption is small. The shortcomings such as long analysis time, low sensitivity and resolution in the analytical methods of HPLC-ELSD, HPCE and HPLC were improved | (Liu et al.) |
|  | Ultra-high performance liquid chromatography-tandem mass spectrometry (UPLC-MS/MS) | Four phenolic acids: chlorogenic acid, ferulic acid, protocatechuic acid and protocatechuic aldehyde | The method is fast and accurate. Due to its advantages of distinguishing according to the difference in mass-to-charge ratio and the detection limit of up to nanogram level, it can overcome the difficulties arising from the HPLC-DAD method in component analysis, such as highly dependent separation, the need to reach microgram level for the detection of substance content, and the need for certain UV absorption of the detection substance | (Bai et al.) |

Table S6 Quality Control of *Prunella vulgaris* L.

| NO. | Detection index | Extraction method of detection index | Detection method | Detection wavelength | Description | Ref. |  |
| --- | --- | --- | --- | --- | --- | --- | --- |
|  | Ursolic acid | Soxhlet extraction method (5h, Ether) | Dual Wavelength TLC-scanning | λs =520 nm; λ_R_ =700 nm | The ursolic acid was specific for the identification of PVL. The average recovery was 98.86%, RSD=1.72% | (Li and Liao) |  |
|  | Rosmarinic acid | Ultrasonic extraction (1h, methanol) | HPLC-PDAD (Phenomenex C_18_) | λ=330 nm | The method is sensitive, accurate, and simple with good repeatability | (Wang et al.) |  |
|  | Ursolic acid, Oleanolic acid | Ultrasonic extraction (2h, 95% ethanol) | RP-HPLC-PDAD (Nucleodur C_18_) | λ=210 nm | The method is simple, accurate, reproducible and suitable for the quality control of PVL | (Zou et al.) |  |
|  | Caffeic acid, Rosmarinic acid | Ultrasonic extraction (1h, 75% ethanol) | HPLC-UVD (Agilent Zorbax C_18_) | λ=330 nm | The method is simple and accurate for the determination of caffeic acid and rosmarinic acid in different parts of PVL | (Zhang et al.) |  |
|  | Ursolic acid, Oleanolic acid and Rosmarinic acid | Soxhlet extraction method (6h, Ether) | HPLC-PDAD (Betasil C_18_) | λ=330 nm | This method is simple, accurate and specific, and the separation effect is better | (Liu and Ding) |  |
|  | Ursolic acid, Oleanolic acid | Soxhlet extraction method (2h, methanol) | Capillary zone electrophoresis | Separation voltage=12.5 KV | The method has the advantages of easy operation, fast separation, high sensitivity, good reproducibility and precision | (Zhang et al.) |  |
|  | Fingerprint of PVL | Ultrasonic extraction (30min, 90% methanol) | HPLC-PDAD (Hypersil C_18_) | λ=208 nm | Fingerprints of 31 batches of PVL were established with 10 common peaks. The method is stable, reliable, simple and reproducible | (Yang et al.) |  |
|  | Ursolic acid; Oleanolic acid; Euscaphic acid; 2α,3α-dihydroxyurs-12en-28oic acid; 2α, 3α,24-trihydroxyolean-12en-28 oic acid | Ultrasonic extraction (30min, ethanol) | HPLC-PDAD (Luna C_18_) | λ=210 nm | The method is simple, high sensitivity, high resolution and short analysis time. It is suitable for the chemical standardization of PVL from different habitats | (Lee et al.) |  |
|  | Rutin; Quercetin | Ethanol extraction (2h *2) | HPLC (Alltima C_18_) | λ=350 nm | The established method was simple and accurate, providing a basis for the quality control of PVL | (Jia) |  |
|  | Caffeic acid; Rosmarinic acid;  Oleanolic acid; Ursolic acid | Ultrasonic extraction (30min, 75% ethanol) | HPLC-PDAD (Elite Sino Chrom ODS-AP) | λ=330 nm; λ =203 nm | The method is sensitive, convenient and accurate, and is suitable for the simultaneous determination of the four constituents in PVL | (Fang et al.) |  |
|  | Rutin; Hyperoside; Quercetin and Kaempferol | Soxhlet extraction method (5h, 90% methanol) | HPLC (Diamonsil-C_18_) | λ=365 nm | This method is simple, accurate, reproducible, and has good separation effect and peak shape without tailing phenomenon. | (Huang et al.) |  |
|  | Oleanolic acid；Ursolic acid；2α，3α-dihydroxy-12 en-28 ursolic acid | Ultrasonic extraction (30min, methanol) | RP-HPLC-PDAD (Diamonsil C_18_) | λ=210 nm | This method is accurate, simple and reproducible. Except from the three triterpenoid acids determined in this experiment, there are four peaks with good separation and shape |  |  |
|  | Fingerprint of PVL | Ultrasonic extraction (1h, 95% methanol) | HPLC-UVD (Diamonsil C18) | λ=210 nm | 29 chromatographic peaks were identified as characteristic peaks of PVL with 8 common peaks. This method is simple, accurate and reproducible | (Qin et al.) |  |
|  | Fingerprint of PVL | Ultrasonic extraction (30min, methanol) | HPLC (Agilent Eclipse XDB-C_18_) | λ=290 nm | Fingerprints of 19 batches of PVL were established with 14 common peaks, could be well separated with high similarity and good precision, repeatability and stability | (Xu et al.) |  |
|  | Fingerprint of PVL | Ultrasonic extraction (30min, methanol) | HPLC-PDAD (Elit Sino Chrom ODS -AP) | λ=203 nm | Fingerprints of 15 batches of PVL with 17 common peaks. This method was advanced, simple, stable, intuitive and representative | (Fang and Lin) |  |
|  | Salviaflaside; Rosmarinic acid | Ultrasonic extraction (30min, methanol) | HPLC (Agilent Eclipse XDB-C_18_) | λ=319 nm | The method is simple, rapid and accurate, and can be used for the determination of the two constituents in PVL | (Lin et al.) |  |
|  | Oleanolic acid; Ursolic acid; Rosmarinci acid and Caffeic acid | Soxhlet extraction method (6h, Ether) | High performance capillary electrophoresis | λ=210 nm; λ=330 nm | This method is simple, rapid, accurate and specific, and can be used to determine the contents of these four active components in PVL | (Guo et al.) |  |
|  | Caffeic acid; Hesperidin; Rosmarinic acid; Quercetin; Luteolin; Oleanolic acid 3-*O*-monoglucuronide; Rhein; Corosolic acid; Oleanolic acid and Ursolic acid | Methanol extraction (2h *2) | HPLC-UV-MS (PDA, RP-Alltima C_18_) | λs =215 nm; λ_R_ =360 nm | Establishment of 14 batches of PVL fingerprints from different areas and identification of 10 common peaks are effective methods for quality control of PVL | (Liu et al.) |  |
|  | Ursolic acid | Ultrasonic extraction (30min, methanol as solvent) | UPLC (ACQUITY UPLC BEH C_18_) | λ=210 nm | The established method for determination of ursolic acid is rapid and accurate with good peak shape and resolution | (Xie et al.) |  |
|  | Total triterpenoids; Total phenolic acids | Ultrasonic extraction (1h, methanol as solvent; 1h, 70% ethanol) | UV spectrophotometer | λ =545 nm; λ =720 nm | The quality of 30 batches of medicinal materials from different areas was evaluated. The method is simple, rapid and easy to popularize | (Yang et al.) |  |
|  | Moisture | Oven-dried method | NIR spectroscpoy | ν= 4486-7408 cm^-1^ | The method is fast, simple, accurate and reproducible | (Lu et al.) |  |
|  | Rosmarinic acid; Maslinic acid; Corosolic acid; Betulin; Oleanolic acid and Ursolic acid | Ultrasonic extraction (1h, 90% ethanol) | HPLC-ELSD (Kromasil 100-5 C_18_) |  | An accurate and reliable method was developed and validated for the first time, and would be helpful in establishing a scientific and rational quality control method for PVL | (Yang et al.) |  |
|  | Luteolin | Ultrasonic extraction (1h, ethanol) | RP-HPLC (Hypersil BDS) | λ =315 nm | This method is simple, rapid, stable, accurate and reproducible, and can be used for quality control of luteolin in PVL | (Fan and Cheng) |  |
|  | Water extracts | Crushing and sieving method | NIR spectroscpoy | ν= 12000-4000 cm^-1^ | A method for determination of water-soluble extract of PVL was established and has the characteristics of no pretreatment, fast detection, no sample destruction and no solvent | (Lu et al., 2016) |  |
|  | Chlorogenic acid; Malic acid; Gallic acid; *p*-Coumaric acid; Caffeic acid; Rosmarinic acid; Protocatechualdehyde; Gluconic acid; Ferulic acid; Umbelliferone; Scopoletin; Esculetin; Cynaroside; Luteolin; Kaempferide; Rutin; Quercetin; Quercitrin; Homoorientin; Hyperoside and Ursolic acid | Ultrasonic extraction (30min, 80% methanol) | HPLC-QTRAP-MS/MS (Syergi Hydro-RP C18) |  | The mathed provides valuable information for revealing the dynamic change laws of metabolite accumulation in PVL and choosing the most suitable harvesting time and genuine producing area of PVL to obtain the best quality | (Liu et al.) |  |
|  | Fingerprint of PVL | Ultrasonic extraction (45min, methanol) | HPLC-UVD (Agilent Eclipse XDB-C_18_) | λ =280 nm | The method for the determination of 12 common peaks in wild and cultivated PVL was established, and the determination time was shortened to 25 min | (Pi et al.) |  |
|  | Fingerprint of PVL | Ultrasonic extraction (30min, ethanol) | UPLC-UVD/PDA (Waters Acquity UPLC HSS T3) | λ =280 nm | UPLC characteristic chromatograms of 16 batches of PVL were studied. 6 common peaks were confirmed, and peak 4 was identified as caffeic acid, and peak 5 was identified as rosmarinic acid. The method is fast, accurate and reliable | (Chen et al.) |  |
|  | Fingerprint of PVL | Reflux extraction (1h, 50 % methanol) | HPLC (Agilent Zorbax C_18_) | λ =210 nm | The fingerprints of 16 batches of Prunella vulgaris were established, and 13 common peaks were calibrated. Five common peaks were identified, including caffeic acid, rutin, hyperoside, rosmarinic acid and ursolic acid. Clustered into 4 groups, 3 principal component factors and 3 differential quality markers | (Huang et al.) |  |

Ao, Z., Chan, M., Ouyang, M. J., Olukitibi, T. A., Mahmoudi, M., Kobasa, D., and Yao, X. (2021). Identification and evaluation of the inhibitory effect of Prunella vulgaris extract on SARS-coronavirus 2 virus entry. PloS one 16(6), e0251649. doi:10.1371/journal.pone.0251649

Bai, Y. B., Li, C., Zhou, Y. M., Pi, S. L., Xia, B. H., Li, Y. M., Lin, L. M., and Liao, D. F. (2015). Chemical constituents of triterpenoids from *Prunella vulgaris* and their antitumor activities. Chinese Traditional and Herbal Drugs 46(26), 3623-3629.

Bai, Y. B., Li, H. Q., Bao, M., and Lin, Y. (2020). Simultaneous Determination of Four Phenolic Acids in Prunella Vulgaris by UPLC-MS/MS. China Pharmaceuticals 29(23), 24-27. doi:10.3969/j. issn.1006-4931. 2020. 23. 006

Byun, S. J., Fang, Z., Jeong, S. Y., Lee, C. S., Son, J. K., and Woo, M. H. (2007). [${\alpha} $]-Amyrin Triterpenoids and Two Known Compounds with DNA Topoisomerase I Inhibitory Activity and Cytotoxicity from the Spikes of Prunella vulgaris var. lilacina. Natural Product Sciences 13(4), 359-364.

Byun, S. J., Lee, J. E., Kim, D. H., Son, J. K., Lee, J. S., Park, Y. S., and Woo, M. H. (Year). "Four $\alpha $-Amyrin Triterpenoids, and their Cytotoxicity and Topoisomerase I Inhibition from the Spikes of Prunella vulgaris", in: *Proceedings of the PSK Conference*: The Pharmaceutical Society of Korea.), 249.242-250.

Cai, F., and Yan, Q. X. (2016). Study on the triterpenoid constituents in herb of *Prunella vulgaris*. Journal of Guangdong Pharmaceutical 32(4), 428-430. doi:10.16809/j.cnki.1006-8783.2016050601

Chen, C., Wu, G., and Zhang, M. (2009). The effects and mechanism of action of Prunella vulgaris L extract on Jurkat human T lymphoma cell proliferation. The Chinese-German Journal of Clinical Oncology 8(7), 426-429. doi:10.1007/s10330-009-0067-x

Chen, J. M. (1988). *Enlightening Primer of Materia Medica.* Beijing, China: People's Medical Publishing House.

Chen, W. F., Li, Z. Y., Wei, M., Sun, D. M., Chen, X. D., Li, L., and Wang, L. W. (2019). Determinaiton of Rosmarinic Acid and Study on UPLC Characteristic Chromatogram of *Prunella vulgaris*. Pharmacy Today 29(9), 613-616. doi:10.12048/j.issn.1674-229X.2019.09.008

Choi, H. G., Kim, T. H., Kim, S. H., and Kim, J. A. (2016). Anti-allergic inflammatory triterpenoids isolated from the spikes of Prunella Vulgaris. Natural product communications 11(1), 31-32. doi:10.1177/1934578X1601100111

Cui, B. L., and Ren, X. J. (2015). Research of the mechanism of MHV-3 induced fulminant hepatic failure in mice and the improvement of total triterpenoid of *prunella vulgaris* L. Chinese Journal of the Frontiers of Medical Science(Electronic Version) 7(10), 133-136.

Deng, J., Li, L., Lin, L. M., Li, Y. M., Xia, B. H., and Liao, D. F. (2021). Metabolic mechanism of *Prunella vulgaris* in treatment of ethanol-induced oxidative stress in rats based on metabonomics. China Journal of Chinese Materia Medica 46(7), 1813-1821. doi:10.19540/j.cnki.cjcmm.20210122.503

Dmitruk, I. S., Dmitruk, S. E., Berezovskaya, T. P., and Prishep, T. P. (1987). Flavones of *Prunella vulgaris*. Chemistry of Natural Compounds 23(3), 374-375.

Dmitruk, S. I. (1986). Coumarins of Prunella vulgaris. Chemistry of Natural Compounds 22(4), 480-480.

Du, B. Q., Zhang, W. X., and Zhang, L. P. (2021a). Anti-tumor Effect of Xiaku Cao( Prunella Vulgaris) on Osteosarcoma Mice by Regulating OPG/RANK/RANKL Signaling Pathway. Acta Chinese Medicine 36(03), 594-600. doi:10.16368/j.issn.1674-8999.2021.03.126

Du, D. S., Cheng, Z. H., and Chen, D. F. (2012). A New Unusual Δ11(12)-Oleane Triterpene and Anti-Complementary Triterpenes from *Prunella Vulgaris* Spikes. Natural Product Communications 7(4), 501-505. doi:10.1177/1934578X1200700422

Du, K., Cao, Y., Liu, B., Xie, L., and Zheng, H. J. (2021b). Effects of Prunella Vulgaris on Apoptosis and Proliferation of Glioma Cell Line U87. Journal of Basic Chinese Medicine 27(9), 1390-1394. doi:10.19945/j.cnki.issn.1006-3250.2021.09.014

Fan, Y., and Cheng, H. L. (2016). Determination of luteolin of Prunella vulgarisLinn from Different by HPLC. World Latest Medicine Information 16(52), 26+30. doi:10.3969/j.issn.1671-3141.2016.52.010

Fang, L., and Lin, N. M. (2012). Quality Assessment of Spica Prunellae by HPLC Fingerprint and Pattern Recognition. Chinese Archives of Traditional Chinese Medicine 30(9), 2034-2037+2147. doi:10.13193/j.archtcm.2012.09.116.fangl.050

Fang, L., Lin, N. M., and Wu, Y. J. (2010). Simultaneous determination of four active components in Spica Prunellae by HPLC. China Journal of Chinese Materia Medica 35(5), 616-619. doi:10.4268 /cjcmm20100517

Fang, Y., Zhang, L., Feng, J., Lin, W., Cai, Q., and Peng, J. (2017). Spica Prunellae extract suppresses the growth of human colon carcinoma cells by targeting multiple oncogenes via activating miR-34a. Oncology reports 38(3), 1895-1901. doi:10.3892/or.2017.5792

Feng, L., Jia, X., Zhu, M., Chen, Y., and Shi, F. (2010a). Chemoprevention by Prunella vulgaris L. Extract of Non-Small Cell Lung Cancer Via Promoting Apoptosis and Regulating the Cell Cycle. Asian Pacific Journal of Cancer Prevention 11(5), 1355-1358.

Feng, L., Jia, X. B., Shi, F., and Chen, Y. (2010b). Identification of two polysaccharides from Prunella vulgaris L. and evaluation on their anti-lung adenocarcinoma activity. Molecules 15(8), 5093-5103. doi:10.3390/molecules15085093

Feng, L., W, O. Y., Xu, Y. H., Wang, S. S., Zhu, Q., and Xiang, P. (2011). Oleanolic acid from Prunella Vulgaris L. induces SPC-A-1 cell line apoptosis via regulation of Bax, Bad and Bcl-2 expression. Asian Pac J Cancer Prev 12(2), 403-408.

Gai, C. Y., Kong, D. Y., and Wang, S. G. (2010). Study on Chemical Constituents of *Prunella vulgaris* L. Chinese Journal of Pharmaceuticals 41(8), 580-582.

Gao, W., Liang, H., Li, Y., Liu, Y., and Xu, Y. (2019). Root extract of Prunella vulgaris inhibits in vitro and in vivo carcinogenesis in MCF-5 human breast carcinoma cells via suppression of angiogenesis, induction of apoptosis, cell cycle arrest and modulation of PI3K/AKT signalling pathway. JBUON, 24(2), 549-554.

Gilsaeng, J. E. O. N. G., Renbo, A. N., Hyunck, P. A. E., Huntaeg, C. H. U. N. G., & Younchul, K. I. M. (2006). Structure-activity relationship of ursane triterpenoids isolated from Prunella vulgaris and Agrimonia pilosa on hemeoxygenase-1 expression. 춘계총회 및 학술대회, 223-224.

Gu, X. J., Li, Y. B., Li, P., Qian, S. H., and Duan, J. A. (2007). Studies on chemical constituents of Prunellavulgaris. China Journal of Chinese Materia Medica 32(10), 923-926.

Gu, X. J., Li, Y. B., Mu, J., and Zhang, Y. (2011). A new phenolic glycoside from Prunella vulgaris. Acta Pharmaceutica Sinica 46(5), 561−563. doi:10.16438/j.0513-4870.2011.05.007

Guo, Q., Qu, H., Zhang, H., and Zhong, X. (2021). Prunella vulgaris L. Attenuates Experimental Autoimmune Thyroiditis by Inhibiting HMGB1/TLR9 Signaling. Drug design, development and therapy 15, 4559-4574. doi:10.2147/DDDT.S325814

Guo, X. H., Wang, Q. W., and Liu, W. (2013). Four Active Component Assay of Xiakucao with High Performance Capillary Electrophoresis. Acta Chinese Medicine 28(1), 69-71. doi:10.16368/j.issn.1674-8999.2013.01.054

He, Y. Q., Li, R. Z., Feng, L. Z., and Li, Z. P. (1985). Studies on the Chemical Constituents of Prunella Vulgaris L. Journal of Peking University(Health Sciences) 17(4), 297-299+320.

Huang, K., Fu, P., Lin, A., and Fan, Y. (2021). Study on HPLC Fingerprint and Chemical Pattern Recognition of Xiakucao ( Prunella vulgaris) from Different Producing Areas. Chinese Archives of Traditional Chinese Medicine 39(12), 124-128+290. doi:10.13193/j.issn.1673-7717.2021.12.029

Huang, Y. Y. (2015). *Yu Qiu Yao Jie.* Beijing, China: People's Medical Publishing House.

Huang, Z. Y., Wang, J. L., Huang, M. J., and Wan, J. F. (2012). Determination of rutin, hyperoside, quercetin and kaempferol in *Prunella vulgaris* by HPLC. Chinese Traditional Patent Medicine 34(3), 520-523.

Hwang, S. M., Kim, J. S., Lee, Y. J., Yoon, J. J., Lee, S. M., Kang, D. G., and Lee, H. S. (2012). Anti-diabetic atherosclerosis effect of Prunella vulgaris in db/db mice with type 2 diabetes. The American journal of Chinese medicine 40(5), 937-951. doi:10.1142/S0192415X12500693

Jia, X. B. (2010). Determination of rutin and quercetin in *Prunella vulgaris* by HPLC. West China Journal of Pharmaceutical Sciences 25(1), 70-71. doi:10.13375/j.cnki.wcjps.2010.01.012

Kim, S. H., Huang, C. Y., Tsai, C. Y., Lu, S. Y., Chiu, C. C., and Fang, K. (2012). The aqueous extract of Prunella vulgaris suppresses cell invasion and migration in human liver cancer cells by attenuating matrix metalloproteinases. The American journal of Chinese medicine 40(03), 643-656. doi:10.1142/S0192415X12500486

Kojima, H., and Ogura, H. (1986). Triterpenoids from Prunella vulgaris. Phytochemistry 25(3), 729-733. doi:10.1016/0031-9422(86)88033-5

Kojima, H., Tominaga, H., Sato, S., and Ogura, H. (1987). Pentacyclic triterpenoids from Prunella vulgaris. Phytochemistry 26(4), 1107-1111. doi:10.1016/S0031-9422(00)82359-6

Kojima, H., Tominaga, H., Sato, S., Takayanagi, H., and Ogura, H. (1988). Two novel hexacyclic triterpenoids from Prunella vulgaris. Phytochemistry 27(9), 2921-2925. doi:10.1016/0031-9422(88)80689-7

Lan, M. Y. (2004). *Materia Medica of South Yunna.* Yunnan, China: Yunnan Science and Technology Press.

Lee, I. K., Kim, D. H., Lee, S. Y., Kim, K. R., Choi, S. U., Hong, J. K., Lee, J. H., Park, Y. H., and Lee, K. R. (2008). Triterpenoic Acids of Prunella vulgaris var. lilacina and Their Cytotoxic Activities In Vitro. Arch Pharm Res 31(12), 1578-1583. doi:10.1007/s12272-001-2154-6

Lee, M. K., Ahn, Y. M., Lee, K. R., Jung, J. H., Jung, O. S., and Hong, J. (2009). Development of a validated liquid chromatographic method for the quality control of Prunellae Spica: determination of triterpenic acids. Analytica Chimica Acta 633(2), 271-277. doi:10.1016/j.aca.2008.12.038

Li, S. Z. (2005). *The Grand Compendium of Materia Medica.* Beijing, China: People's Medical Publishing House.

Li, X. M., and Liao, H. W. (2002). Determination of ursolic acid in *Prunella vulgaris* L. Journal of Guangdong Pharmaceutical University 18(1), 31-32. doi:10.16809/j.cnki.1006-8783.2002.01.012

Li, Y., Ji, B. P., Zheng, J., Li, B., and Zhang, X. F. (2006). Effects of Prunella vulgaris L.Extracts on Blood Glucose and Blood Lipid in Streptozotocin-inducedDiabeticICRMice. Food Science 27(6), 212-215.

Li, Z. X., Xu, J. T., Lin, X. M., Lu, D. Y., Liu, X. H., and Li, G. (2020). Study on the Activity of Prunella vulgaris Aqueous Extract against Zika Virus. Traditional Chinese Drug Research and Clinical Pharmacology 31(12), 1408-1415. doi:10.19378/j.issn.1003-9783.2020.12.004

Liang, J. K., Zhang, L., and Yan, X. M. (2013). ldentification of the Major Chemical Constituents of Prunella Vulgaris L. by HPLC-ESI-MS/MS. Chinese Medicine Modern Dstance Education of China 11(14), 153-154. doi:10.3969/ j issn 1672-2779.2013.14.101

Lin, C. H., Chen, C. S., Wang, Y. C., Lin, E. S., Chang, C. Y., Chen, J. J. Y., and Wan, L. (2021). Fallopia Japonica and Prunella Vulgaris Inhibit Myopia Progression by Suppressing Akt and NFκB Mediated Inflammatory Reactions. Preprints. doi:10.20944/preprints202107.0228.v1

Lin, L. M., Xu, Z. D., Yao, J. X., Liu, J. Y., Li, C., and Wang, Z. M. (2012). Quantitative Analysis of Salviaflaside and Rosmarinic Acid in Prunella vulgaris. Chinese Pharmaceutical Journal 47(15), 1204-1207.

Lin, Y., Yang, C., Tang, J., Li, C., Zhang, Z. M., Xia, B. H., and Liao, D. F. (2020). Characterization and anti-uterine tumor effect of extract from Prunella vulgaris L. BMC Complementary Medicine and Therapies 20(1), 1-11. doi:10.1186/s12906-020-02986-5

Liu, H., Zhong, Y. J., and Wu, D. (2014a). Inhibitory Effect of *Prunella vulgaris* L. Flavonoids on Osteoporosis in Ovariectomized Rats. Modern Food Science and Technology 30(08), 6-11. doi:10.13982/j.mfst.1673-9078.2014.08.014

Liu, J., Feng, L., Gu, J., Wang, R., Zhang, M., Jiang, J., and Zhu, M. (2014b). Simultaneous determination of ten characteristic antioxidant compounds for inhibiting cancer cell proliferation in Prunella vulgaris L. from different regions using HPLC-UV coupled with MS identification. Analytical Methods 6(9), 3139–3146. doi:10.1039/c3ay41754a

Liu, W., and Ding, H. J. (2008). Determination of Ursolic Acid, Oleanolic Acid and Rosmarinic Acid in *Prunella vulgaris* L. by HPLC. Chinese Traditional Patent Medicine 30(4), 577-580.

Liu, Z. X., Hua, Y. J., Wang, S. N., Zou, L. S., Liu, X. H., Zhao, H., and Yan, Y. (2017). Quality evaluation of Prunellae spica based on simultaneous determination of multiple bioactive constituents combined with grey relational analysis. Natural Product Communications 12(7), 1934578X1701200729. doi:10.1177/1934578X1701200729

Lou, H. Y., Yao, J. N., Pan, J., Zheng, S., Liu, Y. Z., Liang, G. Y., and Wei, D. P. (2015). C21 Steroidal Glycosides from Prunella vulgaris. Journal of Carbohydrate Chemistry 34(6), 358-364. doi:10.1080/07328303.2015.1069828

Lu, H. J., Jia, C. C., Cao, Q. X., and Ji, S. G. (2016). Content Determination of Water-soluble Extract in Prunellae Spica by Near-infrared Spectroscopy. Chinese Journal of Experimental Traditional Medical Formulae 22(2), 43-46. doi:10.13422/j.cnki.syfjx.2016020043

Lu, H. J., Jia, C. C., Zhao, H. N., Jiang, B., and Ji, S. G. (2015). Determination of Moisture in Prunella vulgaris L. by Near-infrared Spectroscopy Combined with PLS. Chinese Journal of Pharmaceuticals 46(7), 746-749. doi:10.16522/j.cnki.cjph.2015.07.020

Lu, X. J., Chang, L. M., Chen, T. L., and Liu, B. G. (2022). Study of Efficacy of Prunella Vulgaris Flavonoids on Chondrocyte Apoptosis in Osteoarthritis Rats and lts Action Mechanism. Chinese Journal of Traditional Medical Traumatology & Orthopedics 30(03), 1-6+11.

Meng, Z. M., and He, L. W. (1995). Studies on Constituents of *Prunella vulgaris* L. Journal of China Pharmaeruical University 26(6), 329-331.

Morteza-Semnani, K., Saeedi, M., and Akbarzadeh, M. (2006). The essential oil composition of Prunella vulgaris L. Journal of Essential Oil Bearing Plants 9(3), 257-260. doi:10.1080/0972060X.2006.10643500

Pi, S. L., Hu, Y. Z., Peng, X., Li, Y. M., Lin, L. M., Xia, B. H., and Wu, P. (2017). HPLC Fingerprint Analysis and Pattern Ｒecognition of Wild and Cultivated Prunella vulgaris from Different Habitats. Chinese Pharmaceutical Journal 52(5), 367-371. doi:10.11669 /cpj.2017.05.007

Qi, J., Hu, Z. F., Liu, Z. J., and Yu, B. Y. (2009). Triterpenes from *Prunella vulgaris*. Chinese Journal of Natural Medicines 7(6). doi:10.3724/SP. J. 1009.2009.00421

Qin, W., Ba, Y. Y., Zhang, L. Z., and Shi, R. B. (2012). HPLC fingerprint analysis of Prunella vulgaris from different habitat. China Journal of Traditional Chinese Medicine and Pharmacy 27(5), 1418-1420.

Que, S., Yun , Lou, Y. C., and Hou, J. (2015). Vasodilation Effect and Underlying Mechanism of Active Fraction from Compound *Prunella Vulgaris* L. in Isolated Thoracic Aorta of Rats. China Pharmacist 18(6), 884-887.

Song, J., Zhang, Z., Hu, Y., Li, Z., Wan, Y., Liu, J., and Yang, X. (2021a). An aqueous extract of Prunella vulgaris L. inhibits the growth of papillary thyroid carcinoma by inducing autophagy in vivo and in vitro. Phytotherapy Research 35(5), 2691-2702. doi:10.1002/ptr.7015

Song, Y., Kang, L., Tian, S., Cui, L., Li, Y., Bai, M., and Miao, M. (2021b). Study on the anti-hepatocarcinoma effect and molecular mechanism of Prunella vulgaris total flavonoids. Journal of Ethnopharmacology 273(2021), 113891. doi:10.1016/j.jep.2021.113891

Tabba, H. D., Chang, R. S., and Smith, K. M. (1989). Isolation, purification, and partial characterization of prunellin, an anti-HIV component from aqueous extracts of Prunella vulgaris. Antiviral research 11(5-6), 263-273. doi:10.1016/0166-3542(89)90036-3

Tan, J., Qi, H., and Ni, J. (2015). Extracts of endophytic fungus xkc-s03 from Prunella vulgaris L. spica inhibit gastric cancer in vitro and in vivo. Oncology Letters 9(2), 945-949. doi:10.3892/ol.2014.2722

Tang, S. W. (2011). *Materia Medica Arranged According to Pattern.* Beijing, China: China Medical Science Press

Tao, H. J. (2013). *Miscellaneous Records of Famous Physicians.* Beijing, China: China Press of Traditional Chinese Medicine.

Tian, H. X., Liu, Q. Q., Kang, X., Tian, Y., and Fan, W. P. (2020). Molecular mechanism of *Prunella vulgaris* in alleviating autoimmune hepatitis in mice. Chinese Journal of Cellular and Molecular Immunology 36(7), 590-595. doi:10.13423/j.cnki.cjcmi.009029

Tian, J., Xiao, Z. Y., Chen, Y. Y., Zhao, Y. Y., and Wang, Z. J. (2000). STRUCTURE IDENTIFICATION OF VULGARSAPONIN A. Acta Pharmaceutica Sinica 35(1), 29-31. doi:10.16438/j.0513 -4870.2000.01.008

Wang, A. (2005). *Essentials of Materia Medica.* Beijing, China: People's Medical Publishing House.

Wang, H. B., Zhang, Z. Y., and Su, Z., Wu (1994). The constituents of the essential oil from three plants of Prunella. Chinese Pharmaceutical Journal 29(11), 652-653.

Wang, L., Gao, H. X., Jiang, H. W., Yan, Y. B., Wang, W., and Chai, X. L. (2017). Effect of Xiakucao (Sel fheal ) Combined with Gouteng (Uncaria) on Blood Pressure and Heart in Spontaneous Hypertension Rats. Guiding Journal of Traditional Chinese Medicine and Pharmacy 23(19), 19-22+33. doi:10.13862/j.cnki.cn43-1446/r.2017.19.006

Wang, Q. L., Wang, H., Li, Y. H., Gao, Z. J., and Jia, Y. J. (2021). Effects of prunella vulgaris on the proliferation, invasion and EMT of cervical cancer cells. The Chinese Journal of Clinical Pharmacology 37(20), 2780-2782. doi:10.13699/j.cnki.1001-6821.2021.20.015

Wang, Z. J., Tang, L. Y., Fu, M. H., He, Y., and Fang, J. (2008). Studies on Flavonoids in Prunella vulgaris. Lishizhen Medicine and Materia Medica Research 19(8), 1966-1967.

Wang, Z. J., Zhao, Y. Y., Tu, G. Z., Hong, S. L., and Chen, Y. Y. (1999). Studies on the Chemical Constituents from Prunella Vulgaris. Acta Pharmaceutica Sinica 34(9), 679-681. doi:10.16438/j.0513-4870.1999.09.010

Wang, Z. J., Zhao, Y. Y., Wang, B., Ai, T. M., and Chen, X. Y. (2001). Depsides from Prunella vulgaris. Chinese Journal of Experimental Traditional Medical Formulae (S1).

Wang, Z. J., Zhao, Y. Y., Wang, B., Li, J. X., Ai, T. M., and Chen, Y. Y. (2000). A New Phenylpropanoid and Triterpenoids from Prunella vulgaris. Journal of Chinese Pharmaceutical Sciences 9(3), 128-130.

Wang, Z. J., Zhao, Y. Y., Wang, B., Zhang, Q. Y., Tang, L. Y., and He, Y. (2006). Studies on analytical method of rosmarinic acid in *Spica Prunellae* L. Chinese Journal of Pharmaceutical Analysis 26(3), 399-400.

Wu, H. P., Ha, T. Z., and Gao, M. (2010). Effects of Prunella vulgaris extract on mRNA expression of α-glucosidase, SGLT-1, GLUT-2 and Na ^+^ -K ^+^ -ATPase in Caco-2 cells. Chinese Journal of Biochemical and Pharmaceuticals 31(6), 373-376.

Wu, P. (1996). *Shen Nong’s Classic of the Materia Medica.* Beijing, China: Scientific and Technical Documentation Press.

Wu, X. M., and Xu, Y. P. (2017). Effect of Prunella vulgaris L. on proliferation and apoptosis of cholangiocarcinoma cells. Zhejiang Journal of Traditional Chinese Medicine 52(3), 227-228. doi:10.13633/j.cnki.zjtcm.2017.03.053

Xie, W. J., Cao, Y., Bai, Y. B., Xie, J. C., Xia, B. H., Li, Y. M., Pan, Q. P., and Lin, L. M. (2014). Determination of ursolic acid in Prunella vulgaris L. by UPLC. China Modern Medicine 21(28), 11-13.

Xu, D. C., Liu, S. J., Yu, N. J., and Fang, C. W. (2010a). Study on Chemical Constituents of Spica Prunellae. Modern Chinese Medicine 12(1), 21-22+46. doi:10.13313/j.issn.1673-4890.2010.01.014

Xu, Y., Xu, G., Liu, L., Xu, D., and Liu, J. (2010b). Anti‐invasion effect of rosmarinic acid via the extracellular signal‐regulated kinase and oxidation–reduction pathway in Ls174‐T cells. Journal of cellular biochemistry 111(2), 370-379. doi:10.1002/jcb.22708

Xu, Z. D., Yao, J. X., Liu, J. Y., Li, C., and Lin, L. M. (2012). Study on HPLC Fingerprint of Prunella vulgaris. Journal of Chinese Medicinal Materials 35(8), 1234-1237. doi:10.13863/j.issn1001-4454.2012.08.020

Yan, D., Xie, W. J., Li, C., Bai, Y. B., Lin, L. M., Liao, D. F., Xia, B. H., and Gong, L. M. (2016). Chemical Components from Prunellae Spica and Their Anti-tumor Activities *in Vitro*. Chinese Journal of Experimental Traditional Medical Formulae 22(11), 49-54. doi:10.13422/j. cnki. syfjx. 2016110049

Yan, J. (2008). *Materia Medica of Combinations.* Beijing, China: China Press of Traditional Chinese Medicine.

Yang, A. P., Zheng, Z. G., Liu, F., Liu, J., Wang, R. X., Yang, H., and Liu, H. (2020). Screening for Potential Antibreast Cancer Components From Prunellae Spica Using MCF-7 Cell Extraction Coupled with HPLCESI- MS/MS. Natural Product Communications 15(6), 1-5. doi:10. 1177/ 1934 578X 20931965

Yang, C. (2018). Anti-uterine tumor effect and characterization of volatile oil constituents in Prunella vulgaris L*.* Master's thesis, University of South China.

Yang, C., and Zhao, J. H. (2019). Analysis of Volatile Components from Different Parts of Prunella vulgaris by GP-MSE/GC-MS. Science and Technology of Food Industry 40(13). doi:10.13386/j.issn1002-0306.2019.13.029

Yang, H., Li, J., Guo, W. Y., and Zhao, Y. (2008). Study on HPLC Fingerprint of *Prunella vulgaris* L. Journal of Mathematical Medicine 22(1), 76-78.

Yang, J., Hu, Y. J., Yu, B. Y., and Qi, J. (2016). Integrating qualitative and quantitative characterization of Prunellae Spica by HPLC-QTOF/MS and HPLC-ELSD. Chinese Journal of Natural Medicines 14(5), 391-400. doi:10.3724/SP.J.1009.2016.00391

Yang, J., Qi, J., and Bo, Y. (2015). Determination of Total Triterpenoids and Total Phenolic Acids in Prunellae Spica Collected from Different Places. Strait Pharmaceutical Journal 27(8), 29-31.

Yang, L. J., Li, Z. Q., and Fan, P. (1988). GC/FT-IR Analysis of Essential oil Composition of Prunella vulgaris L. . Chinese Journal of Pharmaceutical Analysis 8(5), 264-266.

Yang, Y., Nan, H., Wang, G., Yang, W., and Xu, J. (2013). Comparative determination of the volatile components of Prunella vulgaris L. from different geographical origins by headspace solid-phase microextraction and gas chromatography-mass spectrometry. Analytical Letters 46(13), 2001-2016. doi:10.1080/00032719.2013.782551

Yang, Y., Tantai, J., Sun, Y., Zhong, C., and Li, Z. (2017). Effect of hyperoside on the apoptosis of A549 human non‑small cell lung cancer cells and the underlying mechanism. Molecular medicine reports 16(5), 6483-6488. doi:10.3892/mmr.2017.7453

Yao, L. (2015). *Materia Medica Arranged by Channel Tropism.* Beijing, China: China Press of Traditional Chinese Medicine.

Yao, X. J., Wainberg, M. A., and Parniak, M. A. (1992). Mechanism of inhibition of HIV-1 infection in vitro by purified extract of Prunella vulgaris. Virology 187(1), 56-62. doi:10.1016/0042-6822(92)90294-Y

Yu, Q., Qi, J., and Liu, S. J. (2012). Study on Chemical Constituents from Prunella vulgaris. Chinese Journal of Experimental Traditional Medical Formulae 18(5), 107-109. doi:10.13422/j.cnki.syfjx.2012.05.039

Yu, Q., Qi, J., Wang, L., Liu, S. J., and Yu, B. Y. (2015). Pentacyclic Triterpenoids from Spikes of Prunella vulgaris L. Inhibit Glycogen Phosphorylase and Improve Insulin Sensitivity in 3T3‐L1 Adipocytes. Phytotherapy research 29(1), 73-79. doi:10.1002/ptr.5228

Zhang, D. H. (2006). Research on the extraction and isolation of the polysaccharide of Prunella vugaris. Journal of Biology 23(3), 39-41.

Zhang, F., Zhu, P. Y., and Song, F. Y. (2008a). Determination of oleanolic acid and ursolic acid in *Prunella vulgaris* L. by capillary electrophoresis with a high frequency conductivity detector. Journal of Guangdong Pharmaceutical University 24(2), 108-111. doi:10.16809/j.cnki.1006-8783.2008.02.004

Zhang, L. Z., Guo, Y. J., Tu, G. Z., Shi, R. B., Qin, W., Ba, Y. Y., and Yang, F. (2008b). A novel triterpenoid saponin from Prunella vulgaris. Acta Pharmaceutica Sinica 43(2), 169-172. doi:10.16438/j.0513 -4870.2008.02.004

Zhang, L. Z., Qin, W., Zhang, X. H., Zhao, C. L., Jiang, Y. Y., and Guo, Y. J. (2007a). Assay method for contents of caffeic acid and rosmarinic acid in the different parts of *Prunella vulgaris* L. Journal of Beijing University of Traditional Chinese Medicine 30(5), 343-345.

Zhang, S. P., Deng, Z. Y., Huang, C., Xie, J. L., He, Y., and Li, J. (2012). Protective effects of total triterpenoids of *prunella vulgaris* L. against carbon tetrachloride-induced acute liver injury in rats. Acta Universitatis Medicinalis Anhui 47(9), 1054-1058. doi:10.19405/j.cnki.issn1000-1492.2012.09.011

Zhang, X., Ao, Z., Bello, A., Ran, X., Liu, S., Wigle, J., and Yao, X. (2016). Characterization of the inhibitory effect of an extract of Prunella vulgaris on Ebola virus glycoprotein (GP)-mediated virus entry and infection. Antiviral research 127, 20-31. doi:10.1016/j.antiviral.2016.01.001

Zhang, Y., But, P. P. H., Ooi, V. E. C., Xu, H. X., Delaney, G. D., Lee, S. H., and Lee, S. F. (2007b). Chemical properties, mode of action, and in vivo anti-herpes activities of a lignin–carbohydrate complex from Prunella vulgaris. Antiviral research 75(3), 242-249. doi:10.1016/j.antiviral.2007.03.010

Zhang, Y. J., and Yang, C. R. (1995). Two New Ursane Glycosides from Prunella Vulgaris in France. Acta Botanica Yunnanica 17(4), 468-472.

Zheng, S. (2012). Study on the anti-tuberculosis constituents of Prunella vulgaris L. Nei Mongol Journal of Traditional Chinese Medicine (14), 33. doi:10.16040/j.cnki.cn15-1101.2012.14.047

Zheng, X. Z., Guo, R., Li, J., Wang, W. T., Zhang, X. D., and Xu, Q. L. (2021). Effects and Mechanism of Prunella vulgaris on Expressions of Survivin and Caspase-3 in Esophageal Cancer Cells. Food and Nutrition in China 27(08), 63-65. doi:10.19870/j.cnki.11-3716/ts.20210715.001

Zhou, Y. M., Tang, J., Xiong, S. H., Li, Y. M., Lin, Y., Xia, B. H., Lin, L. M., and Liao, D. F. (2017). Polar Chemical Constituents from Prunella vulgaris L. and Their Anti-human Breast Tumor Activities. Chinese Pharmaceutical Journal 52(5), 362-366.

Zou, S. Q., Jiang, B. Q., and Chen, W. (2007). Detemination of Two Triterpene Acids in *Prunella vulgaris* L. by RP-HPLC. Lishizhen Medicine and Materia Medica Research 18(1), 5-6.
